# Supplementary material for: The impact of surveillance and control on highly pathogenic avian influenza outbreaks in poultry in Dhaka division, Bangladesh
Source: PLoS Comput Biol. 2018 Sep 13;14(9):e1006439. doi: 10.1371/journal.pcbi.1006439 (PMC6155559; doi:10.1371/journal.pcbi.1006439)
Supplement: S1 Supporting Information — This supplement consists of the following parts: (1) Overview of existing and prospective response protocols and contingency plans for control of H5N1 HPAI in Bangladesh; (2) Description of the H5N1 poultry epidemiological data for Bangladesh; (3) Supplementary figures; (4) Supplementary tables. (PDF) [file pcbi.1006439.s001.pdf]

Supporting Information: The impact of surveillance and control on Highly Pathogenic Avian Influenza outbreaks in poultry in Dhaka division, Bangladesh.

Edward M. Hill Thomas House Madhur S. Dhingra Wantanee Kalpravidh
Subhash Morzaria Muzaffar G. Osmani Eric Brum Mat Yamage
Md. A. Kalam Diann J. Prosser John Y. Takekawa Xiangming Xiao
Marius Gilbert Michael J. Tildesley

Table of Contents

1 Response protocols and contingency plans for control of HPAI H5N1 in Bangladesh . . . 1
1.1 Pre-existing strategies . . . 1
1.2 Prospective strategy . . . 3

2 Bangladesh H5N1 poultry epidemiological data . . . 4

3 Supplementary figures . . . 6

4 Supplementary tables . . . 17

1 Response protocols and contingency plans for control of HPAI H5N1 in Bangladesh

1.1 Pre-existing strategies

In Bangladesh, response protocols towards H5N1 HPAI outbreaks in poultry have previously been based on short-term response actions, intended to stop and eliminate a newly introduced pathogen. Accordingly, the focus of HPAI control programs has been on case detection, identification of premises deemed to be in direct contact with a premises reporting infection, and subsequent stamping out of flocks with reported infection [1].

We provide below an overview of pre-existing H5N1 HPAI response protocols in Bangladesh (both historical and current), split by intervention type.

Culling

Prior to 2008, Bangladesh adopted a ring culling approach to combat HPAI outbreaks. Specifically, poultry flocks within 1km of premises with confirmed HPAI infection were designated to be culled.

Nowadays, in the case of a commercial poultry premises reporting HPAI infection, just the poultry flock on that premises is culled (i.e. focal culling). If HPAI infection is found in a backyard farm poultry flock, either culling takes place solely at that farm, or the flock at the infected farm is culled along with backyard farm poultry flocks within 200m/300m if they also report mortality (M.A. Kalam, personal communication).

Vaccination

Prior to 2012, vaccination against HPAI was not considered.

From 2012 onwards, vaccines against HPAI have been available for use on commercial layer and breeder farms. This began with a pilot prophylactic vaccination programme being carried out in two districts in 2012. The government has subsequently expanded the programme, upping its scope to ten districts (M.G. Osmani and M.A. Kalam, personal communication).

Active surveillance

From 2008 to 2012, Bangladesh ran a small-scale active surveillance system. The active surveillance system comprised of teams of community health workers across the country, each monitoring specified farms and reporting to livestock officers mortality events and the presence of any clinical signs of disease. The scheme was discontinued in 2013 for monetary reasons (M.G. Osmani and M.A. Kalam, personal communication).

However, there have subsequently been surveillance schemes established within Bangladesh that are ongoing, demonstrating the capability to carry out policies of this nature. The surveillance based schemes currently in operation may be split into three categories:

(1) Environmental sampling: Environmental sampling is being used to monitor the situation within live bird markets [2].

(2) Trial village surveillance programme: FAO supported trial surveillance programmes comprise enrolled villages being surveyed twice a week, with deployment of rapid detection tests if HPAI viruses are suspected (M.A. Kalam, personal communication).

(3) Upazila-to-Community programme: The DLS (Department of Livestock Services) and FAO supported Upazila-to-Community (U2C) programme is an initiative that avails veterinary services to rural communities to improve livestock production and disease control, increasing resilience to emerging disease events.

U2C was initially implemented in nine pilot upazilas (subdistricts), then expanded to 126 more with plans to eventually cover all 496 upazilas in Bangladesh.

The U2C programme, by reaching out to communities, bridges the inherent bias in traditional surveillance systems by including control of diseases in the extensive livestock production system. Additionally, the U2C initiative has enabled the DLS to develop capacity in participatory disease surveillance, community outreach, and principles of avian influenza control and prevention (M.A. Kalam and E. Brum, personal communication).

1.2 Prospective strategy

The information within this subsection, outlining a revised strategy for control of H5N1 HPAI in Bangladesh, has been provided by E. Brum and M.S. Dhingra (personal communication).

Strategy overview

A multi-stakeholder approach is being used to develop a H5N1 HPAI disease control strategy for Bangladesh that integrates biosecurity measures at all levels of poultry production, effective vaccination, disease surveillance, outbreak management, and risk reduction measures along the live bird marketing chain. The proposed strategy is designed to achieve continuous risk mitigation efforts for progressive control of an endemic disease.

Operational goals

Operational goals of the proposed strategy are to decrease the prevalence of H5N1 by protecting poultry in farms and villages, securing farms, and reducing spread of H5N1. Emphasis will be placed on biosecurity and preventative vaccination as a cost-effective means of achieving progressive control.

Objectives

Envisage the operational goal being attained via the delivery of four primary objectives:

(1) Biosecurity measures to be implemented at all farm levels as appropriate: Secure farms from entry of pathogens through implementation of effective biosecurity.

(2) Preventative vaccination: Increase effective preventative vaccination in layer and breeder farms by ensuring use of the correct vaccine (antigenically potent and matched with circulating local strain antigenic characterization), the correct schedule and correct techniques (farmers using vaccine correctly, training, and awareness).

(3) Outbreak management: Given the widespread and endemic nature of the disease, community-level farmer engagement by both the public and private sectors is essential. Involves progressively expanding the DLS-FAO U2C initiative nationally and motivating communities to report poultry mortality and/or morbidity events immediately (within 24 hours) to complement the surveillance system for early disease detection and reporting of sick/dead birds.

(4) Clean poultry transports: Reduce spread of pathogens from collector yards (wholesale markets) to farms.

Relevance of response actions designed to eliminate a newly introduced pathogen

The aim of protecting poultry in farms means areas of intensive commercial poultry farms would be making serious efforts to stay free from HPAI. It is important to stress that the proposed policy does not preclude the interventions of interest in this work, as they remain of practical use in combating newly-surfacing outbreaks in regions whose commercial poultry premises attain H5N1 HPAI virus-free status.

2 Bangladesh H5N1 poultry epidemiological data

From 2007 to 2012 inclusive there have been 554 poultry premises with reported H5N1 infection in Bangladesh. These were predominately commercial premises (497 cases), with 57 cases reported from backyard flocks. The Bangladesh office of FAO/UN provided a dataset of confirmed infected premises up to June 2011. Cases occurring after June 2011 were obtained from the OIE World Animal Health Information Database (WAHID) Interface [3]. For the case data provided by the latter source, we were informed that the Department of Livestock Services reported regularly to WAHID regarding HPAI outbreaks in Bangladesh, with this usually occurring within 24 hours according to the code of the World Organisation for Animal Health (M.G. Osmani, personal communication). We therefore presumed WAHID contained all reported Bangladesh HPAI event information.

For each infected premises the data documented its spatial location, the date that infection was reported, the date of culling and the total number of poultry infected and culled. We divided the infected premises data into distinct epidemic waves. These were estimated by looking for significant gaps between premises infection dates, with a gap of two months or more used to signify the end of a wave and the start of a new one. The dates and number of premises reporting infection for each wave are displayed in Table A.

Table A: Breakdown of H5N1 HPAI poultry epidemic waves in Bangladesh.

	Start month	End month	Reported cases	Birds culled
**Wave 1**	March 2007	July 2007	55	248,271
**Wave 2**	September 2007	May 2008	232	1,254,141
**Wave 3**	November 2008	June 2009	37	56,047
**Wave 4**	January 2010	June 2010	31	193,114
**Wave 5**	January 2011	May 2011	161	617,773
**Wave 6**	November 2011	April 2012	26	133,480

Start month, end month, number of reported infected premises and birds culled in each of the H5N1 poultry epidemic waves in Bangladesh. In addition to the cases within each wave listed above, the following reported cases occurred between waves: one case in September 2008; two cases in August 2009; one case in June 2001; four cases in August-September 2011; four cases in October 2012 - March 2013.

There were 52 poultry premises recorded as being infected that were not part of the 2010 premises census. When analysing a specific wave, out of the additional premises entries we included only those premises that were infected during that wave (including the reported backyard farm cases when applicable). The transmission models used here were fitted to the two largest epidemic waves, wave 2 and wave 5, with reported case epidemiological curves for these waves presented in Figure A.

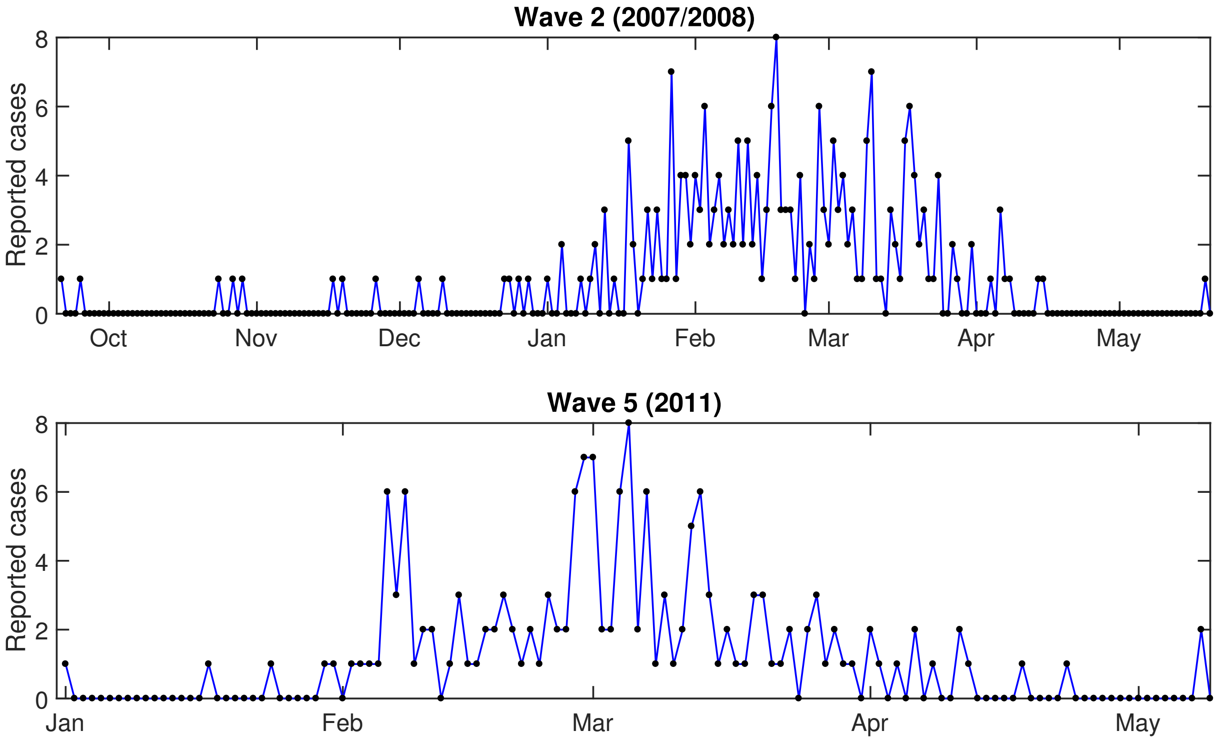

Figure A: Epidemiological curve of premises reporting H5N1 influenza infection in Bangladesh. Black dots correspond to counts of newly reported infected poultry premises per day in Bangladesh during epidemic waves 2 and 5 respectively.

3 Supplementary figures

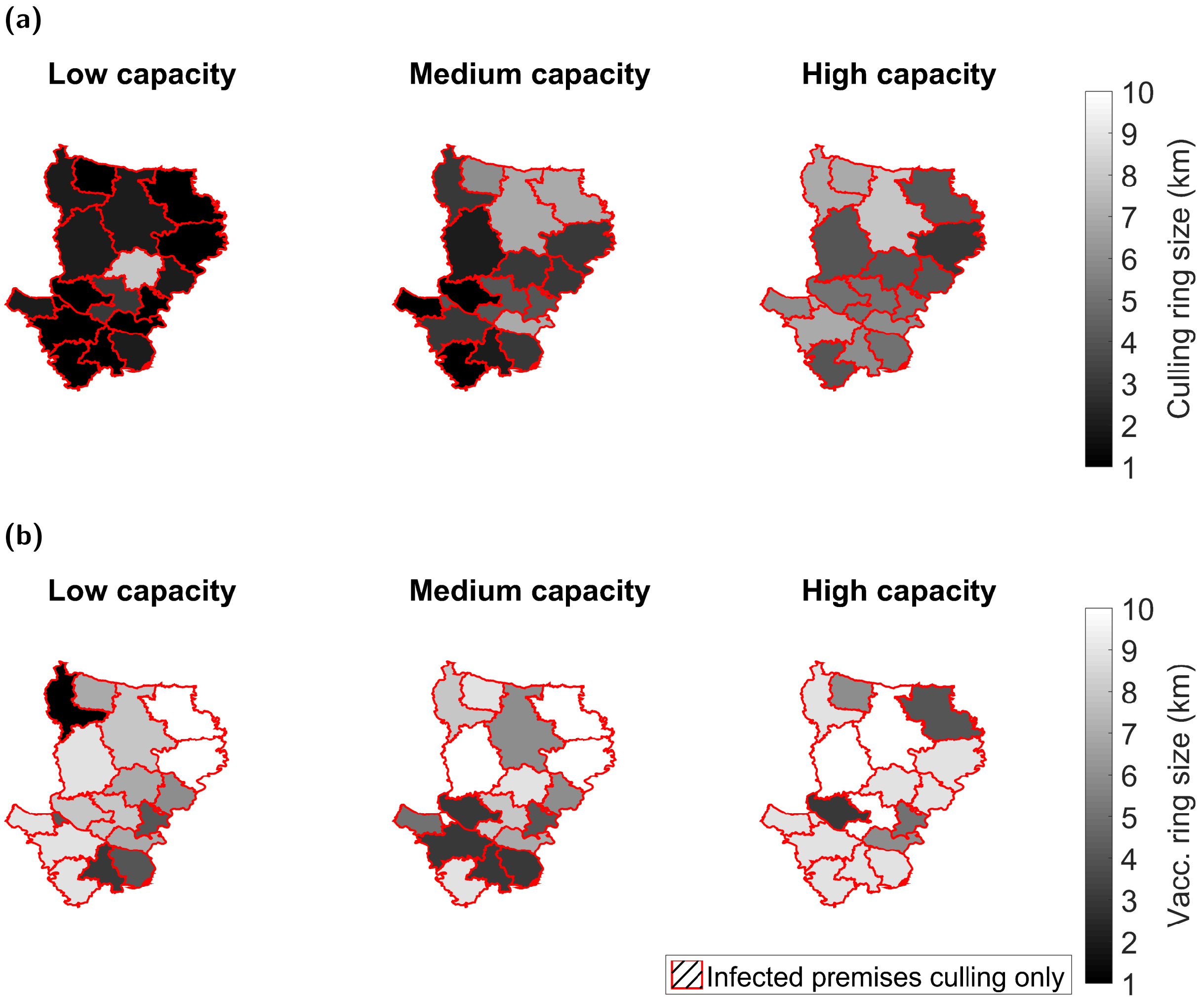

Figure B: Maps displaying the ring range that optimises minimising the average outbreak duration with respect to district of outbreak origin and control capacity level, under wave 2 type transmission dynamics. For each combination of control capacity level, district of outbreak origin and control type 1,000 simulation runs were performed. Hatching of a district indicates the preferred strategy was culling infected premises only, while solid shading corresponds to the ring size determined as the optimal severity of response against outbreaks that originally emerged in that district. Lighter shading corresponds to a larger intervention region. Types of control tested were **(a)** ring culling, and **(b)** ring vaccination. For full results see Table D.

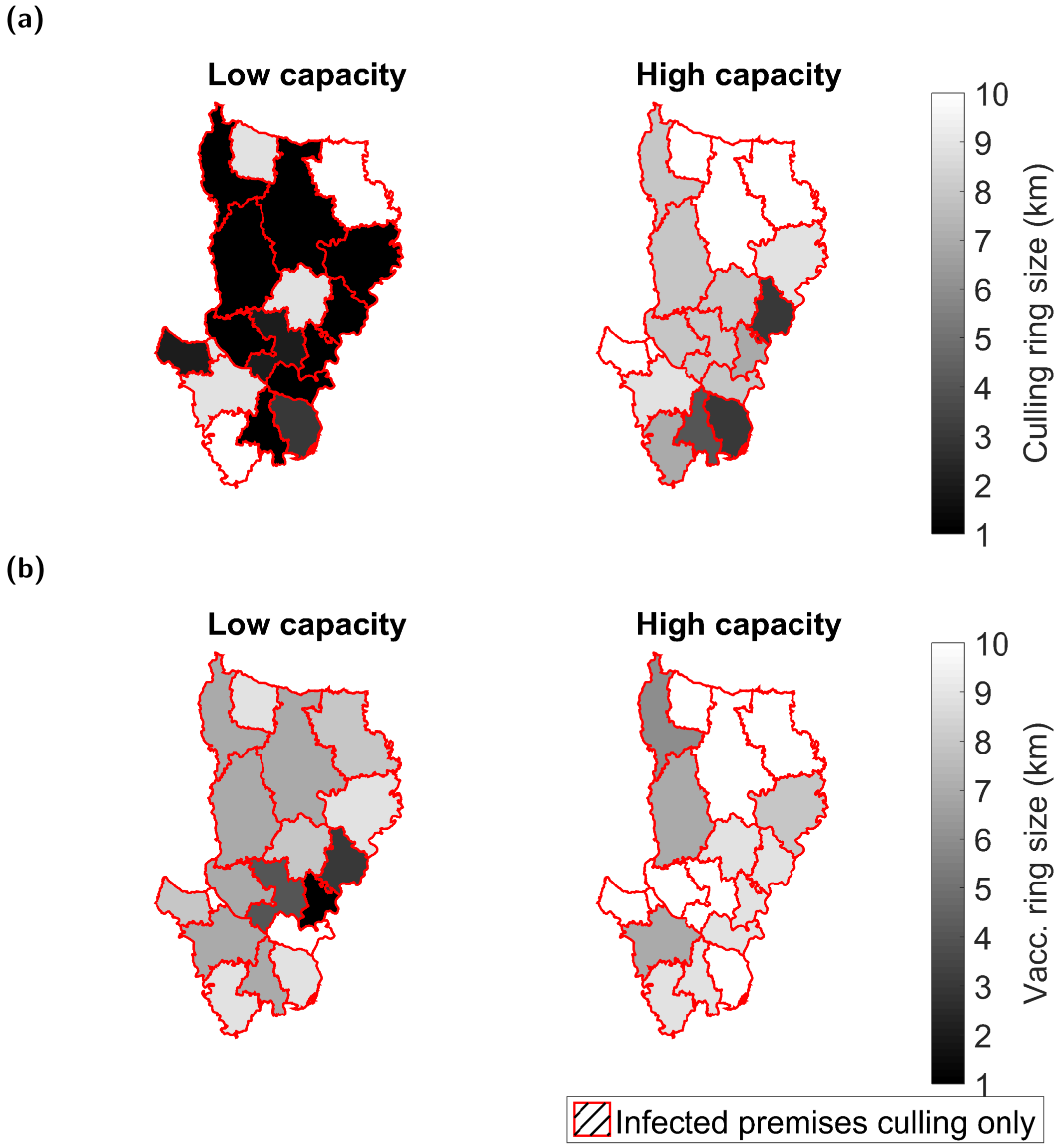

Figure C: Maps displaying the ring range that optimises minimising outbreak duration with respect to district of outbreak origin and control capacity level, under wave 5 type transmission dynamics. For each combination of control capacity level, district of outbreak origin and control type 1,000 simulation runs were performed. Hatching of a district indicates the preferred strategy was culling infected premises only, while solid shading corresponds to the ring size determined as the optimal response against outbreaks that originally emerged in that district. Lighter shading corresponds to a larger intervention region. Types of control tested were **(a)** ring culling, and **(b)** ring vaccination. For full results see Table F.

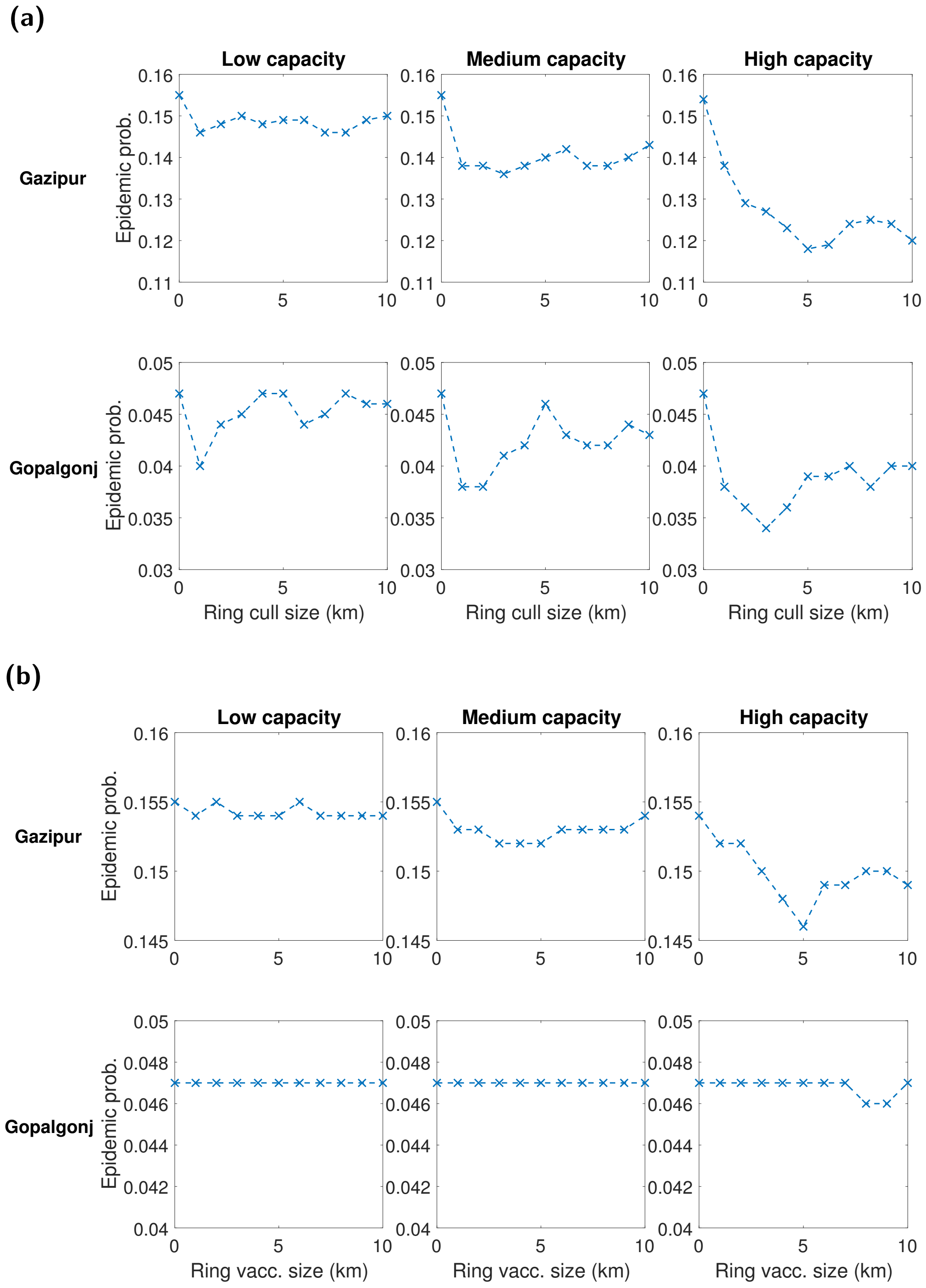

Figure D: Sensitivity of epidemic probability to intervention ring size and capacity restrictions, under wave 2 transmission dynamics. For outbreaks originating in the Gazipur and Gopalgonj districts (for a district locator map see Figure 1), the panels show predicted epidemic probability with the following intervention measure utilised: **(a)** ring culling; **(b)** ring vaccination. In all panels a ring size of 0km corresponds to a control action of culling infected premises only. Larger variations in this control metric are observed across the range of ring culling sizes tested compared to ring vaccination. Analogous outcomes were found for outbreaks seeded in the remaining districts.

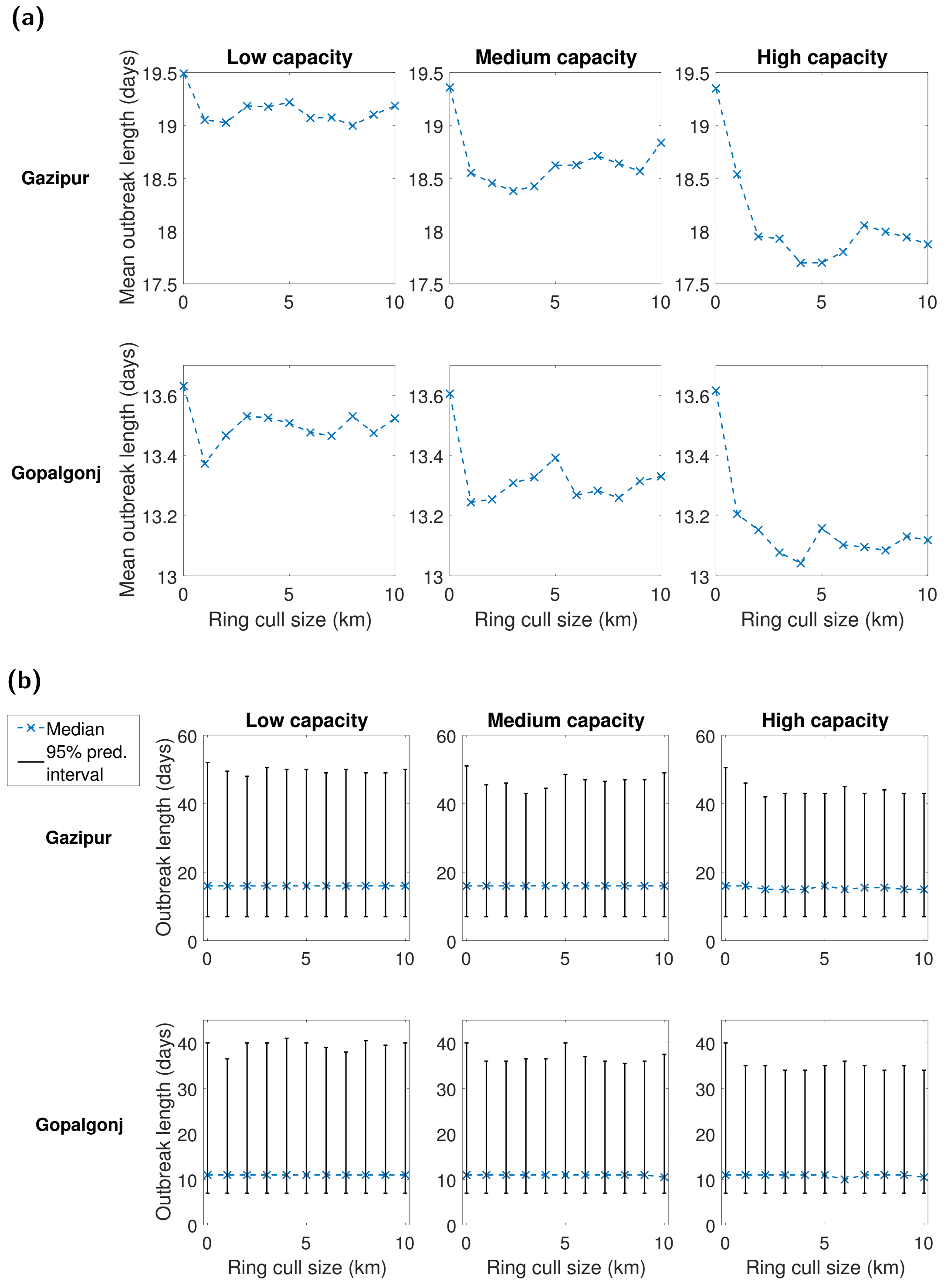

Figure E: Sensitivity of outbreak duration to culling ring size and capacity restrictions, under wave 2 transmission dynamics. For outbreaks originating in the Gazipur and Gopalgonj districts (for a district locator map see Figure 1), the panels are as follows: **(a)** Average outbreak duration against culling ring size under each capacity level, with larger variations evident under less restrictive capacity levels; **(b)** 95% prediction intervals (black bars) for outbreak duration against culling ring size under each capacity level, with median values depicted by the blue markers and dashed line. Similar ranges are obtained, independent of capacity level and culling severity. Note that in all panels a ring size of 0km corresponds to a control action of culling infected premises only. Analogous outcomes were found for outbreaks seeded in the remaining districts. For each combination of intervention method and district of outbreak origin 1,000 simulation runs were performed.

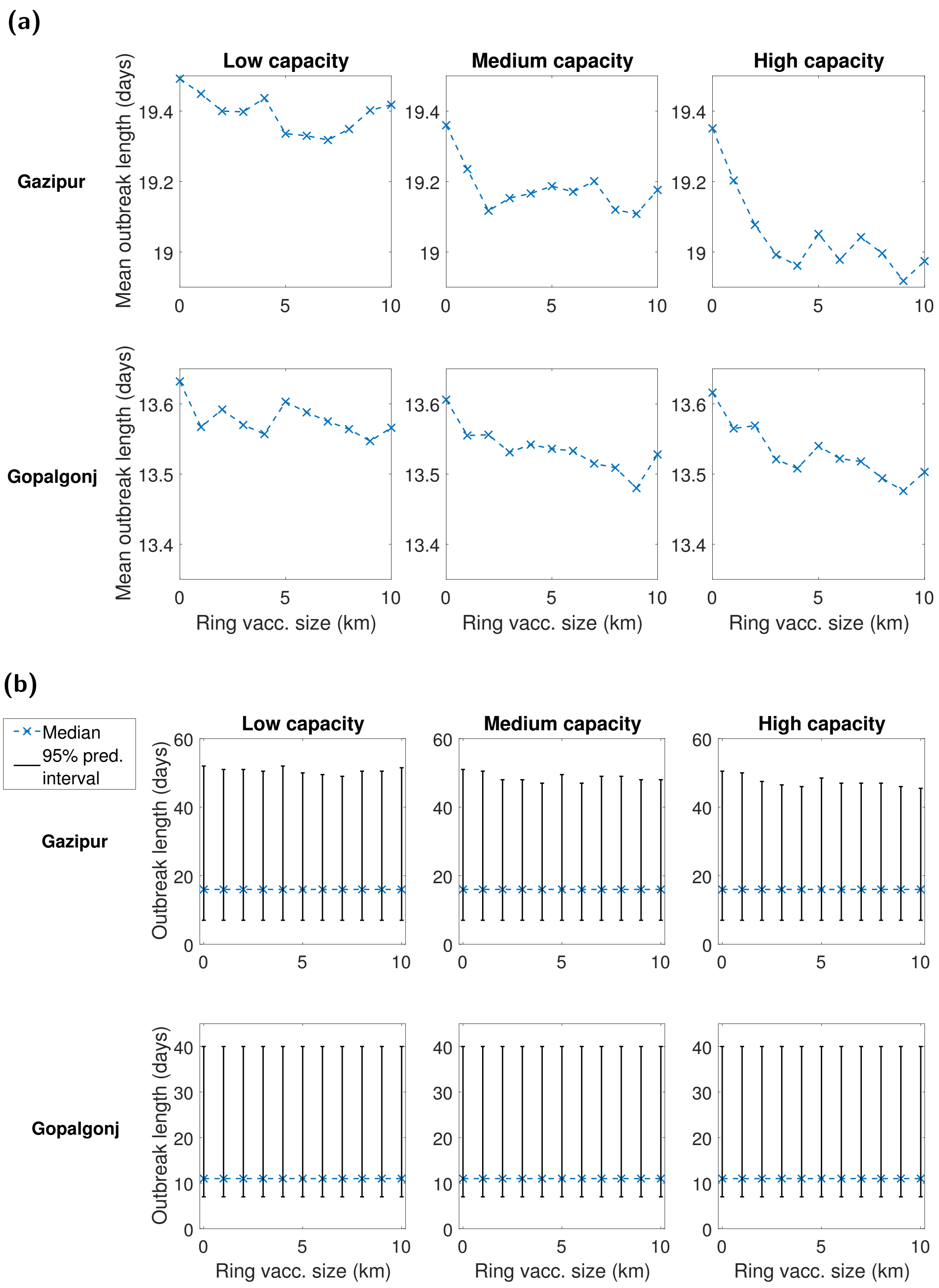

Figure F: Sensitivity of outbreak duration to vaccination ring size and capacity restrictions, under wave 2 transmission dynamics. For outbreaks originating in the Gazipur and Gopalgonj districts (for a district locator map see Figure 1), the panels are as follows: **(a)** Average outbreak duration against vaccination ring size under each capacity level, with larger variations evident under less restrictive capacity levels; **(b)** 95% prediction intervals (black bars) for outbreak duration against vaccination ring size under each capacity level, with median values depicted by the blue markers and dashed line. Similar ranges are obtained, independent of capacity level and vaccination severity. Note that in all panels a ring size of 0km corresponds to a control action of culling infected premises only. Analogous outcomes were found for outbreaks seeded in the remaining districts. For each combination of intervention method and district of outbreak origin 1,000 simulation runs were performed.

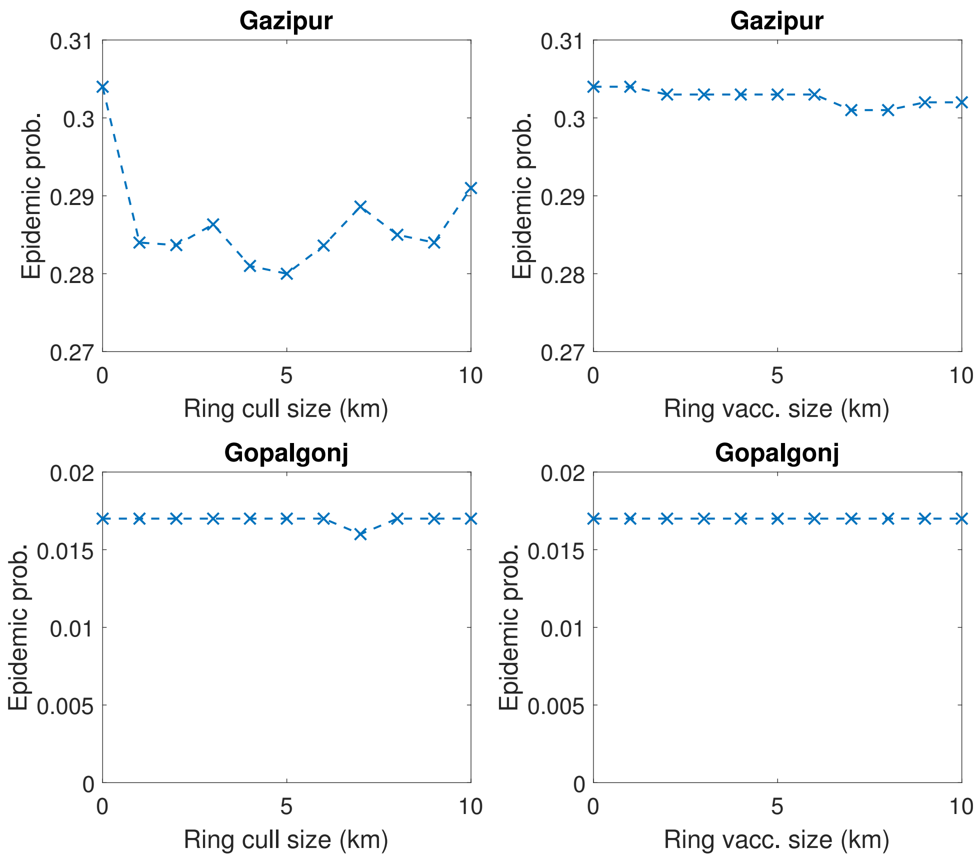

Figure G: Sensitivity of epidemic probability to vaccination ring size with high capacity constraints in place, under wave 5 transmission dynamics. Predicted epidemic probabilities against intervention ring size for outbreaks originating in the Gazipur and Gopalgonj districts (for a district locator map see Figure 1), exposing the minor variations in this control metric across the suite of ring sizes tested. Note that in all panels a ring size of 0km corresponds to a control action of only culling those premises reporting infection. Analogous outcomes were found for outbreaks seeded in the remaining districts.

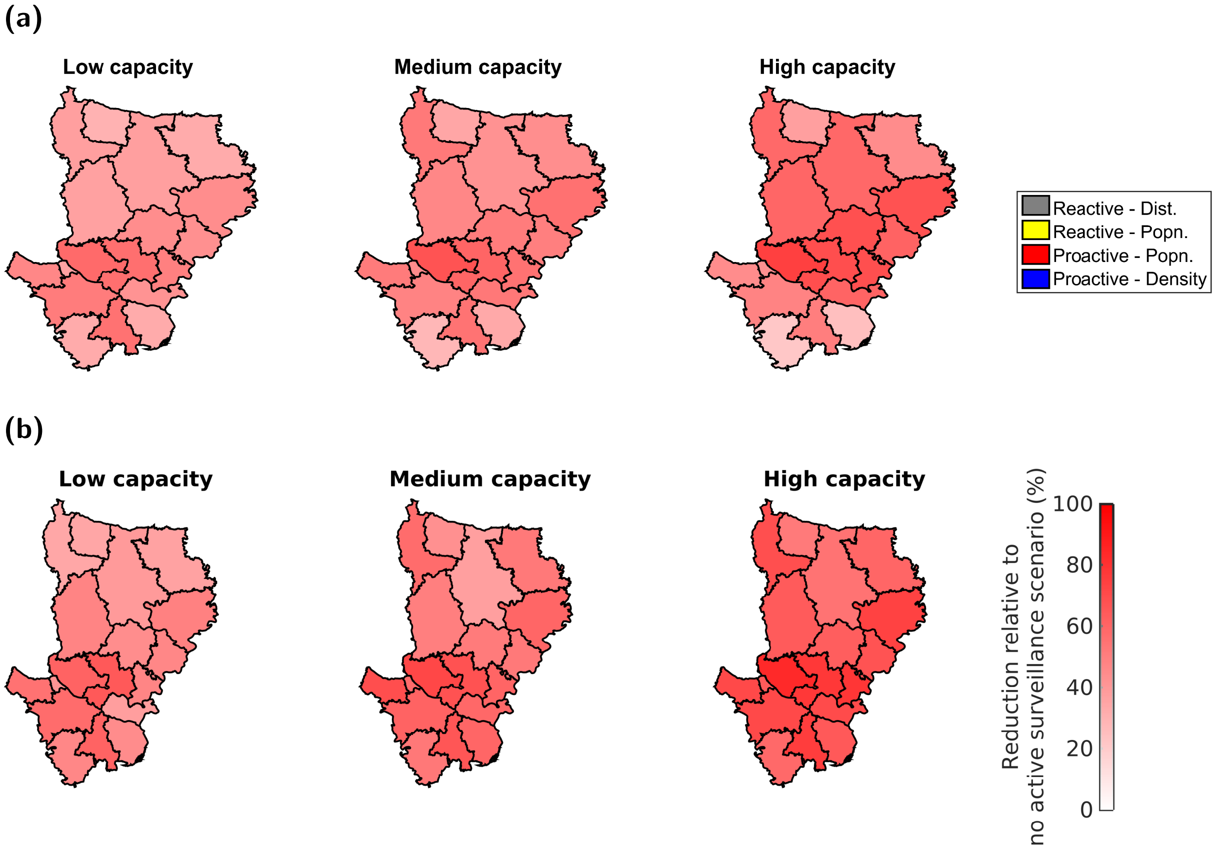

Figure H: Maps displaying the preferred active surveillance strategy to optimise control objectives with respect to district of outbreak origin and capacity setting, for outbreaks with wave 5 type transmission dynamics. For each combination of active surveillance method and district of outbreak origin 500 simulation runs were performed. District colour corresponds to the active surveillance strategy determined to be optimal for countering outbreaks originating from that district (grey - 'reactive by distance', yellow - 'reactive by population', red - 'proactive by population', blue - 'proactive by premises density'). Transparency coincides with the reduction in the objective metric relative to the scenario where no active surveillance was utilised, with completely transparent corresponding to a 0% reduction (no improvement) and completely opaque corresponding to a 100% reduction. The 'proactive by population' scheme was found to be preferred in all cases when optimising either control objective. **(a)** Minimising average outbreak duration, **(b)** minimising the probability of an epidemic. For full results see Tables I and J.

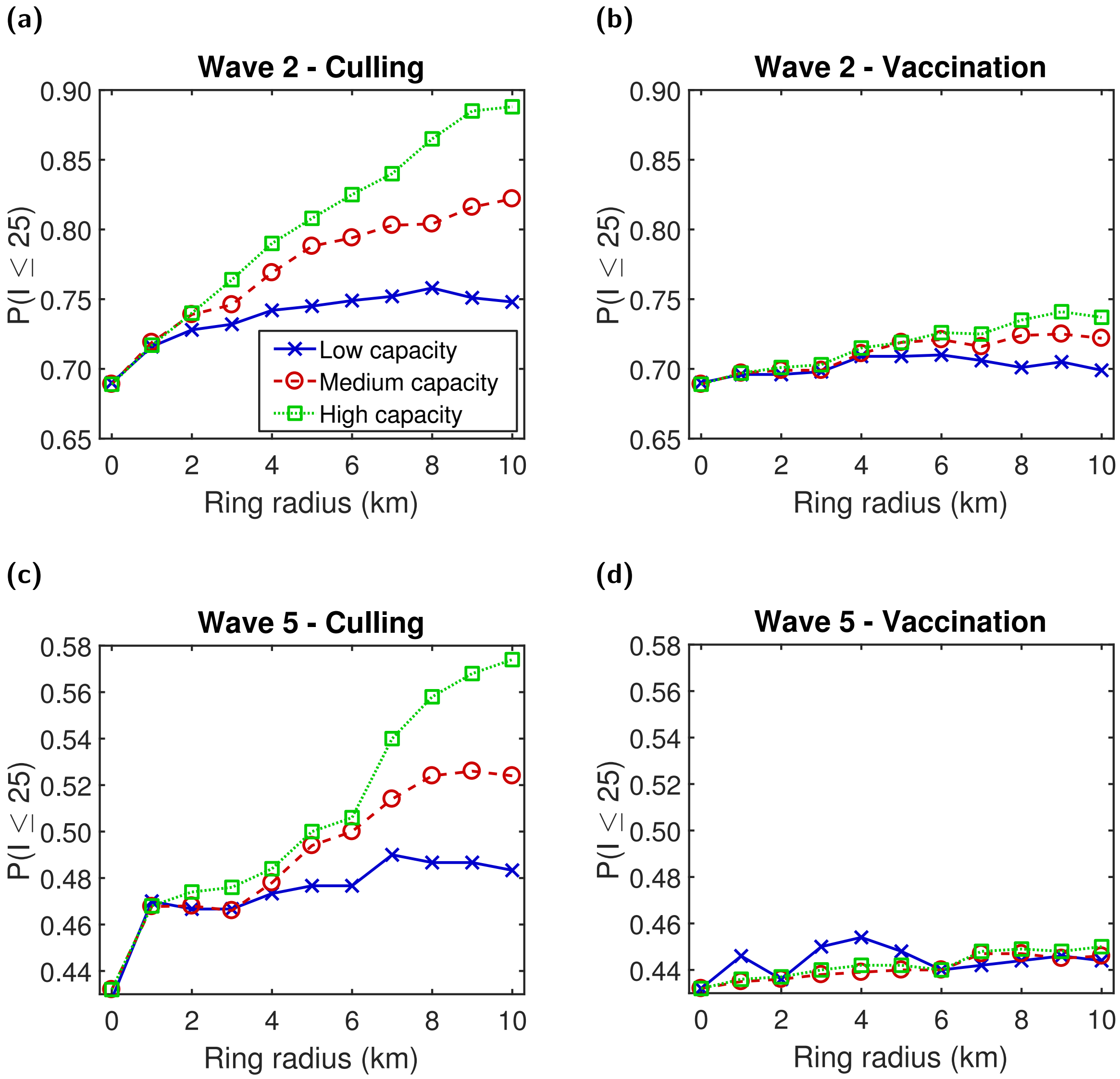

Figure I: Predicted probability of outbreak size (I) not exceeding 25 premises for different ring culling and vaccination radii. For each transmission model and control method combination, the three capacity settings of interest, low (solid blue line, crosses), medium (dashed red line, circles), and high (dotted green line, squares) displayed disparate behaviour. **(a)** Wave 2 - culling; **(b)** wave 2 - vaccination; **(c)** wave 5 - culling; **(d)** wave 5 - vaccination. In all panels a ring size of 0km corresponds to a control action of only culling those premises reporting infection. Results are averaged over 1,000 simulations and 500 simulations for wave 2 and wave 5 type transmission dynamics respectively.

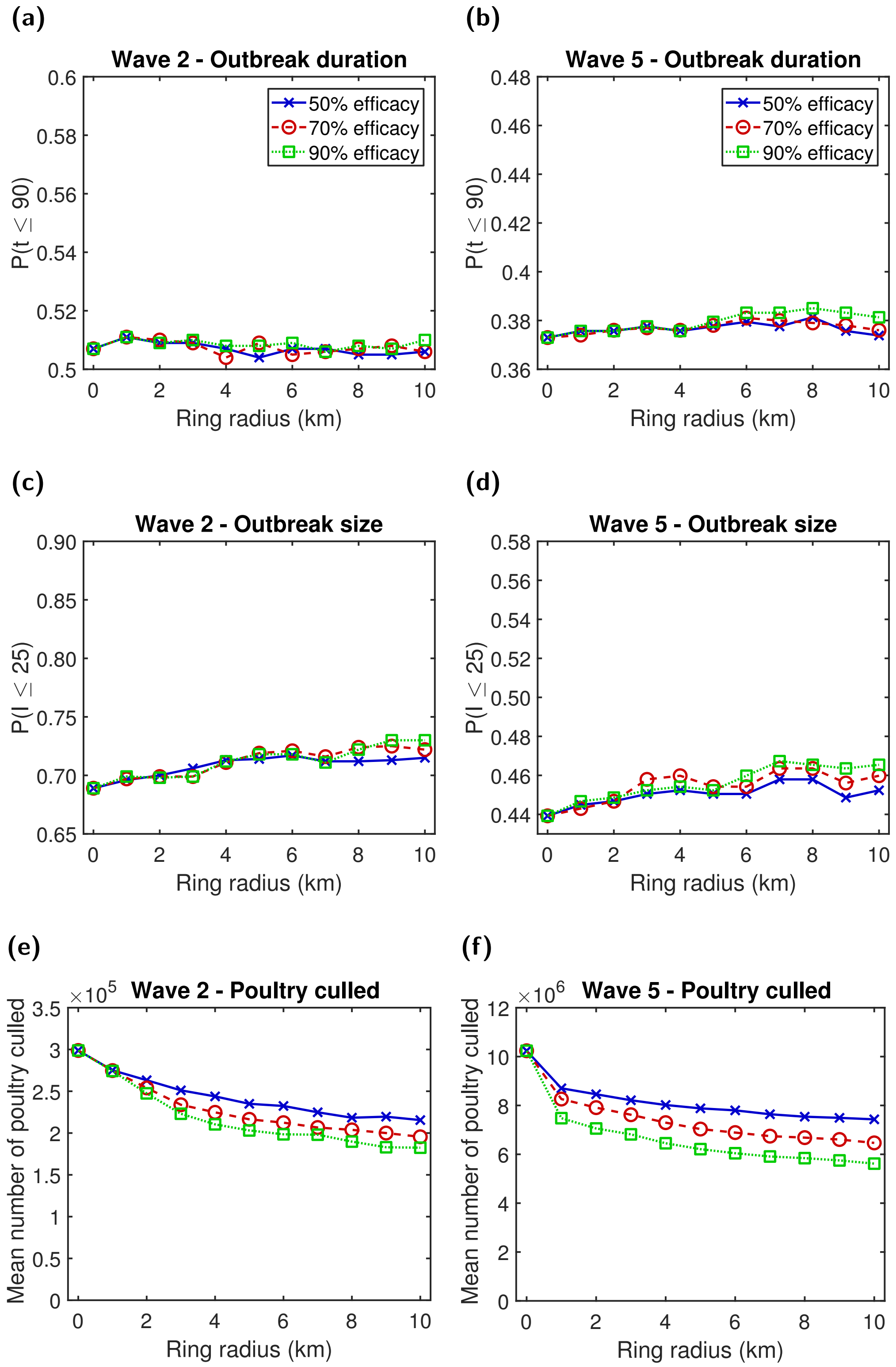

Figure J: Sensitivity of specific control objective metrics to vaccine efficacy. For each combination of transmission model, ring vaccination radii and control objective metric, the three vaccine efficacies tested were 50% (solid blue line, crosses), 70% (dashed red line, circles), and 90% (dotted green line, squares). The vaccine effectiveness delay was fixed at seven days, with medium-level capacity constraints in place. Variations in outcomes are apparent for the expected number of poultry culled control objective. **(a)** Wave 2 - outbreak duration of 90 days or less; **(b)** wave 5 - outbreak duration of 90 days or less; **(c)** wave 2 - epidemic size of 25 premises or less; **(d)** wave 5 - epidemic size of 25 premises or less; **(e)** wave 2 - average number of poultry culled; **(f)** wave 5 - average number of poultry culled. In all panels a ring size of 0km corresponds to a control action of only culling those premises reporting infection. Results are averaged over 1,000 simulations and 500 simulations for wave 2 and wave 5 type transmission dynamics respectively.

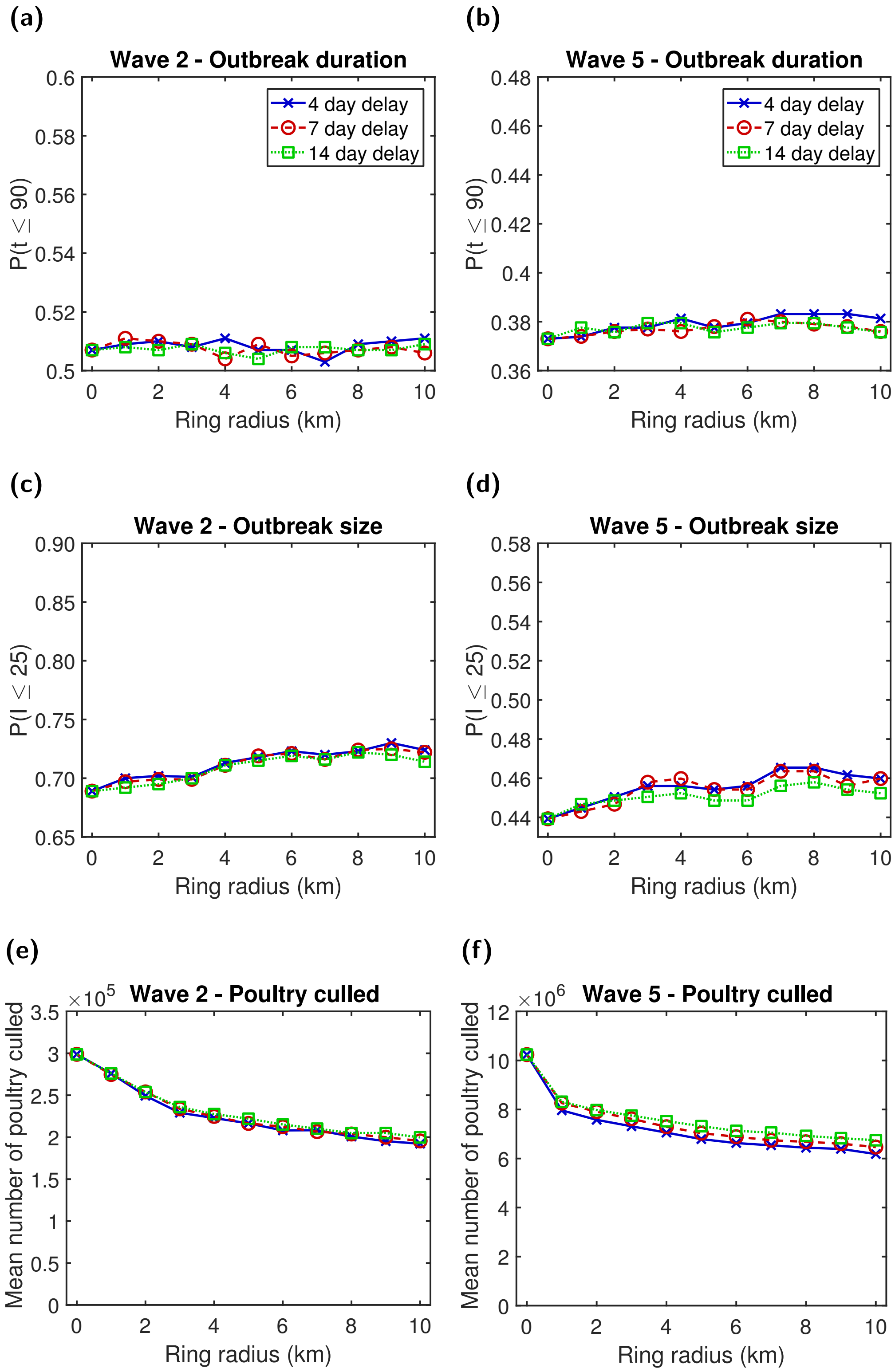

Figure K: Sensitivity of specific control objective metrics to delay in vaccine effectiveness. For each combination of transmission model, ring vaccination radii and control objective metric, the three vaccine effectiveness delay times tested were four days (solid blue line, crosses), seven days (dashed red line, circles), and 14 days (dotted green line, squares). Vaccine efficacy was fixed at 70%, with medium-level capacity constraints in place. **(a)** Wave 2 - outbreak duration of 90 days or less; **(b)** wave 5 - outbreak duration of 90 days or less; **(c)** wave 2 - epidemic size of 25 premises or less; **(d)** wave 5 - epidemic size of 25 premises or less; **(e)** wave 2 - average number of poultry culled; **(f)** wave 5 - average number of poultry culled. In all panels a ring size of 0km corresponds to a control action of only culling those premises reporting infection. Results are averaged over 1,000 simulations and 500 simulations for wave 2 and wave 5 type transmission dynamics respectively.

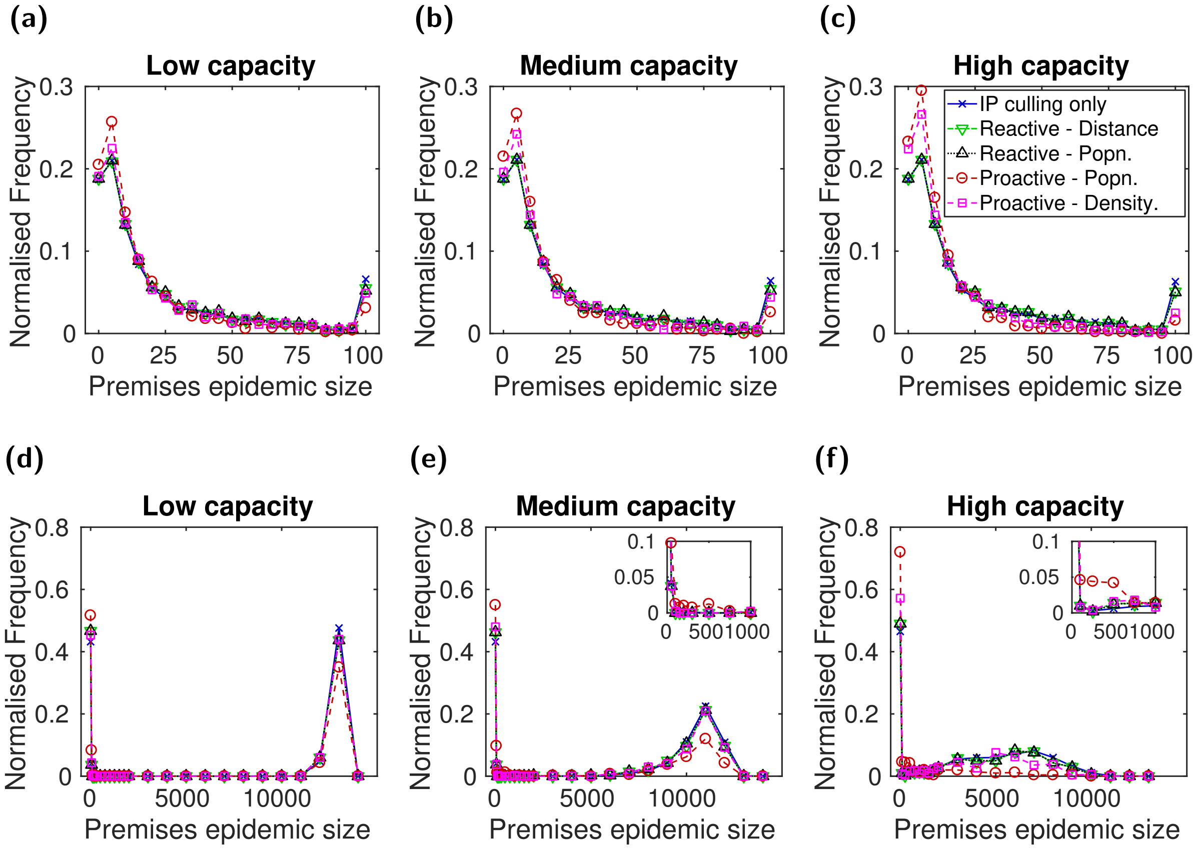

Figure L: Impact on premises epidemic size using different active surveillance strategies with the specified capacity restriction. Four premises targeting strategies were tested and compared to the case where no active surveillance was in place (solid blue line, crosses); 'reactive by distance' (dash-dot green line, inverted triangles), 'reactive by population' (dotted black line, triangles), 'proactive by population' (dashed red line, circles), 'proactive by premises density' (dashed magenta line, squares). The proactive strategies led to a decreased chance of an outbreak reaching more than 100 premises, while the reactive strategies offer minor gains compared to having no active surveillance. **(a–c)** Wave 2 transmission model, where the normalised frequency at 100 also includes all epidemic sizes 100 or greater. **(d–f)** Wave 5 transmission model.

4 Supplementary tables

Table B: Parameter summary statistics for preferred H5N1 influenza transmission models fitted to two historical poultry epidemics within the Dhaka division, Bangladesh.

		Wave 2	Wave 5
$\mathbf{t_c}$	Mean	1.06×10^{-7}	1.71×10^{-10}
	Median	7.70×10^{-8}	1.56×10^{-10}
	(95% CI)	$(7.29 \times 10^{-9}, 3.78 \times 10^{-7})$	$(5.86 \times 10^{-11}, 3.63 \times 10^{-10})$
$\boldsymbol{\epsilon}$	Mean	4.11×10^{-6}	1.04×10^{-5}
	Median	3.98×10^{-6}	1.02×10^{-5}
	(95% CI)	$(1.42 \times 10^{-6}, 7.91 \times 10^{-6})$	$(5.02 \times 10^{-6}, 1.72 \times 10^{-5})$
$\boldsymbol{\alpha}$	Mean	-0.358	0.0136
	Median	-0.345	0.0136
	(95% CI)	(-0.666, -0.159)	(-0.122, 0.143)
**p**	Mean	1.06	1.05
	Median	1.06	1.05
	(95% CI)	(0.923, 1.19)	(0.826, 1.26)
**q**	Mean	0.0574	1.06
	Median	0.0427	1.06
	(95% CI)	(0.00175, 0.189)	(0.844, 1.28)

Parameter mean, median and 95% credible intervals (CI) from 1,000 samples obtained from MCMC.

Table C: Epidemic probabilities for wave 2 type transmission dynamics under top performing scheme within each intervention type, stratified by the district where the outbreak originated. For each combination of capacity setting and district of outbreak origin (seed district), we present the epidemic probabilities when only culling infected premises (IP cull), alongside the top performing schemes for optimising this control objective within each of the three simulated intervention types (ring culling, ring vaccination, active surveillance). The strategy that optimised the objective (minimising the probability of an epidemic) is highlighted in bold. Epidemic probabilities were obtained by averaging over 1000 simulation runs. We use — to denote intervention types with no schemes that outperformed a policy of only culling infected premises. All probabilities are given to 3 d.p., with percentages given to 2 s.f.

	Seed district		Intervention type							
**Capacity**	**ID**	**Name**	**IP cull**	**Ring cull**		**Ring vacc.**		**Active surv.**		**Red.**
**Low**	1	Jamalpur	0.065	1km	0.058	2km	0.063	**C**	**0.012**	82%
	2	Sherpur	0.055	1km	0.047	2km	0.053	**C**	**0.010**	82%
	3	Nasirabad	0.103	2km	0.096	2km	0.101	**C**	**0.023**	78%
	4	Netrakona	0.065	1km	0.0057	2km	0.063	**C**	**0.012**	82%
	5	Tangail	0.099	1km	0.093	9km	0.096	**C**	**0.024**	76%
	6	Gazipur	0.155	1km	0.146	1km	0.154	**C**	**0.039**	75%
	7	Kishoreganj	0.110	1km	0.102	5km	0.109	**C**	**0.022**	80%
	8	Narshingdi	0.138	2km	0.129	8km	0.136	**C**	**0.040**	71%
	9	Manikgonj	0.096	1km	0.087	1km	0.095	**C**	**0.018**	81%
	10	Dhaka	0.111	3km	0.104	8km	0.109	**C**	**0.030**	73%
	11	Naray Angonj	0.122	6km	0.117	—	—	**C**	**0.041**	66%
	12	Munshigonj	0.077	2km	0.071	2km	0.076	**C**	**0.018**	77%
	13	Rajbari (west)	0.058	1km	0.051	2km	0.056	**C**	**0.011**	81%

Continued on next page

Table C: Continued from previous page

	Seed district		Intervention type							
**Capacity**	**ID**	**Name**	**IP cull**	**Ring cull**		**Ring vacc.**		**Active surv.**		**Red.**
	14	Rajbari (east)	0.063	1km	0.056	2km	0.061	**C**	**0.013**	79%
	15	Faridpur	0.058	1km	0.051	2km	0.056	**C**	**0.009**	84%
	16	Gopalgonj	0.047	1km	0.040	—	—	**C**	**0.008**	83%
	17	Madaripur	0.056	1km	0.048	2km	0.055	**C**	**0.005**	91%
	18	Shariatpur	0.056	2km	0.051	2km	0.054	**C**	**0.009**	84%
**Medium**	1	Jamalpur	0.065	2km	0.052	2km	0.063	**C**	**0.007**	89%
	2	Sherpur	0.055	1km	0.044	6km	0.052	**C**	**0.006**	89%
	3	Nasirabad	0.102	7km	0.082	6km	0.097	**C**	**0.011**	89%
	4	Netrakona	0.065	1km	0.053	2km	0.063	**C**	**0.006**	91%
	5	Tangail	0.099	5km	0.085	9km	0.096	**C**	**0.014**	86%
	6	Gazipur	0.155	3km	0.136	3km	0.152	**C**	**0.024**	85%
	7	Kishoreganj	0.110	3km	0.094	9km	0.107	**C**	**0.013**	88%
	8	Narshingdi	0.138	3km	0.121	8km	0.135	**C**	**0.025**	82%
	9	Manikgonj	0.096	1km	0.079	3km	0.094	**C**	**0.011**	89%
	10	Dhaka	0.110	5km	0.095	7km	0.107	**C**	**0.019**	83%
	11	Naray Angonj	0.122	4km	0.108	2km	0.120	**C**	**0.021**	83%
	12	Munshigonj	0.076	1km	0.067	2km	0.074	**C**	**0.008**	89%
	13	Rajbari (west)	0.058	2km	0.046	2km	0.056	**C**	**0.006**	90%
	14	Rajbari (east)	0.063	2km	0.052	9km	0.060	**C**	**0.005**	92%
	15	Faridpur	0.058	2km	0.045	2km	0.056	**C**	**0.005**	91%
	16	Gopalgonj	0.047	1km	0.038	—	—	**C**	**0.003**	94%
	17	Madaripur	0.056	2km	0.045	3km	0.054	**C**	**0.003**	95%
	18	Shariatpur	0.056	2km	0.046	3km	0.053	**C**	**0.004**	93%
**High**	1	Jamalpur	0.065	2km	0.048	9km	0.061	**C**	**0.001**	98%
	2	Sherpur	0.055	2km	0.039	8km	0.051	**C**	**0.002**	96%
	3	Nasirabad	0.102	8km	0.074	7km	0.095	**C**	**0.002**	98%
	4	Netrakona	0.065	3km	0.048	7km	0.061	**C**	**0.001**	98%
	5	Tangail	0.099	5km	0.079	7km	0.096	**C**	**0.002**	98%
	6	Gazipur	0.154	5km	0.118	5km	0.146	**C**	**0.005**	97%
	7	Kishoreganj	0.109	5km	0.083	9km	0.102	**C**	**0.004**	96%
	8	Narshingdi	0.137	5km	0.108	10km	0.129	**C**	**0.008**	94%
	9	Manikgonj	0.096	6km	0.070	6km	092	**C**	**0.001**	99%
	10	Dhaka	0.110	7km	0.086	10km	0.102	**C**	**0.002**	98%
	11	Naray Angonj	0.121	7km	0.094	2km	0.120	**C**	**0.003**	98%
	12	Munshigonj	0.076	7km	0.061	5km	0.073	**C**	**0.001**	99%
	13	Rajbari (west)	0.058	2km	0.044	8km	0.053	**C**	**0.001**	98%
	14	Rajbari (east)	0.063	10km	0.049	10km	0.058	**C**	**0.001**	98%
	15	Faridpur	0.058	2km	0.044	6km	0.055	**C**	**0.001**	98%
	16	Gopalgonj	0.047	3km	0.034	8km	0.046	**C**	**0.001**	98%
	17	Madaripur	0.056	2km	0.041	3km	0.054	**C**	**0.001**	98%
	18	Shariatpur	0.056	3km	0.042	3km	0.053	**C**	**0.001**	98%

Active surveillance strategy legend:
A - reactive by distance,
B - reactive by population,
C - proactive by population,

D - proactive by premises density,
Red. - Percentage decrease in probability of an epidemic using preferred intervention scheme compared to solely culling premises reporting infection.

Table D: Average outbreak duration for wave 2 type transmission dynamics under top performing scheme within each intervention type, stratified by the district where the outbreak originated. For each combination of capacity setting and district of outbreak origin (seed district), we present the average outbreak duration when only culling infected premises (IP cull), alongside the top performing schemes for optimising this control objective within each of the three simulated intervention types (ring culling, ring vaccination, active surveillance). The strategy that optimised the objective (minimising the expected outbreak duration) is highlighted in bold. The mean duration values displayed were obtained by averaging over 1,000 simulation runs. All duration values are given to 1 d.p. in units of days, with percentages given to 2 s.f.

	**Seed district**		**Intervention type**							
**Capacity**	**ID**	**Name**	**IP cull**	**Ring cull**		**Ring vacc.**		**Active surv.**		**Red.**
**Low**	1	Jamalpur	14.9	2km	14.8	1km	14.9	**C**	**11.4**	24%
	2	Sherpur	14.5	1km	14.2	7km	14.5	**C**	**11.3**	22%
	3	Nasirabad	17.0	2km	16.6	8km	16.9	**C**	**12.3**	28%
	4	Netrakona	14.6	1km	14.3	10km	14.6	**C**	**11.5**	22%
	5	Tangail	16.8	2km	16.4	9km	16.7	**C**	**12.4**	26%
	6	Gazipur	19.5	8km	19.0	7km	19.3	**C**	**13.4**	31%
	7	Kishoreganj	17.1	1km	16.6	10km	17.0	**C**	**12.5**	27%
	8	Narshingdi	18.7	2km	18.2	6km	18.6	**C**	**13.8**	27%
	9	Manikgonj	16.7	1km	16.2	8km	16.6	**C**	**11.8**	29%
	10	Dhaka	17.6	3km	17.1	8km	17.4	**C**	**12.7**	28%
	11	Naray Angonj	18.1	1km	17.5	4km	18.0	**C**	**13.5**	26%
	12	Munshigonj	16.0	1km	17.6	7km	15.9	**C**	**12.0**	25%
	13	Rajbari (west)	14.3	2km	14.1	9km	14.3	**C**	**11.0**	23%
	14	Rajbari (east)	14.9	1km	14.7	4km	14.8	**C**	**11.4**	24%
	15	Faridpur	14.4	1km	14.2	9km	14.3	**C**	**10.9**	24%
	16	Gopalgonj	13.6	1km	13.4	9km	13.5	**C**	**10.6**	22%
	17	Madaripur	14.3	1km	14.0	3km	14.2	**C**	**10.5**	26%
	18	Shariatpur	14.3	2km	14.0	4km	14.2	**C**	**11.1**	22%
**Medium**	1	Jamalpur	14.9	3km	15.8	8km	14.9	**C**	**10.2**	32%
	2	Sherpur	14.5	6km	14.1	9km	14.4	**C**	**10.5**	28%
	3	Nasirabad	17.0	7km	16.2	6km	16.8	**C**	**11.4**	33%
	4	Netrakona	14.6	7km	14.1	10km	14.5	**C**	**10.6**	28%
	5	Tangail	16.8	2km	16.1	10km	16.6	**C**	**11.4**	32%
	6	Gazipur	19.4	3km	18.4	9km	19.1	**C**	**12.2**	37%
	7	Kishoreganj	17.0	3km	16.2	10km	16.9	**C**	**11.1**	35%
	8	Narshingdi	18.7	3km	17.7	6km	18.4	**C**	**12.9**	31%
	9	Manikgonj	16.7	1km	16.0	3km	16.6	**C**	**10.5**	37%
	10	Dhaka	17.5	4km	16.6	8km	17.2	**C**	**11.6**	34%
	11	Naray Angonj	18.0	4km	16.9	4km	17.8	**C**	**12.0**	33%
	12	Munshigonj	15.9	7km	15.3	7km	15.8	**C**	**10.3**	35%
	13	Rajbari (west)	14.3	1km	13.9	5km	14.3	**C**	**10.2**	29%
	14	Rajbari (east)	14.9	4km	14.4	10km	14.8	**C**	**10.3**	31%
	15	Faridpur	14.4	3km	14.0	3km	14.3	**C**	**10.2**	29%
	16	Gopalgonj	13.6	1km	13.2	9km	13.5	**C**	**9.9**	27%

Continued on next page

Table D: Continued from previous page

	Seed district		Intervention type							
**Capacity**	**ID**	**Name**	**IP cull**	**Ring cull**		**Ring vacc.**		**Active surv.**		**Red.**
	17	Madaripur	14.2	2km	13.7	3km	14.1	**C**	**9.8**	31%
	18	Shariatpur	14.3	3km	13.8	3km	14.1	**C**	**10.3**	28%
**High**	1	Jamalpur	14.9	7km	14.2	9km	14.8	**C**	**8.4**	44%
	2	Sherpur	14.5	7km	13.7	6km	14.4	**C**	**9.2**	36%
	3	Nasirabad	16.9	8km	15.7	10km	16.7	**C**	**9.9**	42%
	4	Netrakona	14.6	4km	13.8	4km	14.5	**C**	**9.1**	37%
	5	Tangail	16.8	4km	15.8	10km	16.6	**C**	**9.5**	44%
	6	Gazipur	19.4	4km	17.7	9km	18.9	**C**	**9.9**	49%
	7	Kishoreganj	17.0	3km	15.8	9km	16.7	**C**	**8.8**	48%
	8	Narshingdi	18.7	4km	17.0	9km	18.3	**D**	**10.0**	46%
	9	Manikgonj	16.7	5km	15.5	3km	16.5	**C**	**8.2**	51%
	10	Dhaka	17.5	5km	16.0	10km	17.1	**C**	**9.2**	47%
	11	Naray Angonj	18.0	5km	16.1	5km	17.6	**C**	**9.8**	46%
	12	Munshigonj	15.9	6km	14.9	6km	15.7	**C**	**8.1**	49%
	13	Rajbari (west)	14.3	6km	13.6	9km	14.2	**C**	**9.1**	37%
	14	Rajbari (east)	14.8	7km	14.1	9km	14.7	**C**	**9.1**	39%
	15	Faridpur	14.4	7km	13.6	9km	14.2	**C**	**9.0**	37%
	16	Gopalgonj	13.6	4km	13.0	9km	13.5	**C**	**8.8**	35%
	17	Madaripur	14.2	6km	13.5	9km	14.0	**C**	**8.5**	40%
	18	Shariatpur	14.2	5km	13.5	9km	14.1	**C**	**9.2**	35%

Active surveillance strategy legend:
A - reactive by distance,
B - reactive by population,
C - proactive by population,
D - proactive by premises density,
Red. - Percentage decrease in average outbreak duration using preferred intervention scheme compared to solely culling premises reporting infection.

Table E: Epidemic probabilities for wave 5 type transmission dynamics under top performing scheme within each intervention type, stratified by the district where the outbreak originated. For each combination of capacity setting and district of outbreak origin (seed district), we present the epidemic probabilities when only culling infected premises (IP cull only), alongside the top performing schemes for optimising this control objective within each of the three simulated intervention types (ring culling, ring vaccination, active surveillance). The strategy that optimised the objective (minimising the probability of an epidemic) is highlighted in bold. Epidemic probabilities were obtained by averaging over a minimum of 500 simulation runs. We use — to denote intervention types with no schemes that outperformed a policy of only culling infected premises. All probabilities are given to 3 d.p., with percentages given to 2 s.f.

	Seed district		Intervention type							
**Capacity**	**ID**	**Name**	**IP cull**	**Ring cull**		**Ring vacc.**		**Active surv.**		**Red.**
**Low**	1	Jamalpur	0.095	1km	0.094	—	—	**C**	**0.058**	39%
	2	Sherpur	0.046	—	—	—	—	**C**	**0.030**	35%
	3	Nasirabad	0.161	1km	0.155	—	—	**C**	**0.100**	38%
	4	Netrakona	0.055	1km	0.054	—	—	**C**	**0.035**	36%
	5	Tangail	0.138	1km	0.130	1km	0.137	**C**	**0.088**	36%

Continued on next page

Table E: Continued from previous page

	Seed district		Intervention type								
**Capacity**	**ID**	**Name**	**IP cull**	**Ring cull**		**Ring vacc.**		**Active surv.**		**Red.**
	6	Gazipur	0.317	1km	0.299	8km	0.306	**C**	**0.176**	44%
	7	Kishoreganj	0.162	1km	0.159	—	—	**C**	**0.090**	44%
	8	Narshingdi	0.135	1km	0.127	1km	0.134	**C**	**0.076**	44%
	9	Manikgonj	0.192	1km	0.179	—	—	**C**	**0.080**	58%
	10	Dhaka	0.179	1km	0.166	1km	0.177	**C**	**0.075**	58%
	11	Naray Angonj	0.172	1km	0.157	1km	0.170	**C**	**0.091**	47%
	12	Munshigonj	0.083	1km	0.079	4km	0.082	**C**	**0.047**	43%
	13	Rajbari (west)	0.049	1km	0.046	—	—	**C**	**0.022**	55%
	14	Rajbari (east)	0.045	1km	0.042	—	—	**C**	**0.026**	42%
	15	Faridpur	0.059	1km	0.056	—	—	**C**	**0.025**	58%
	16	Gopalgonj	0.017	—	—	—	—	**C**	**0.009**	47%
	17	Madaripur	0.059	1km	0.056	—	—	**C**	**0.023**	61%
	18	Shariatpur	0.020	1km	0.018	—	—	**C**	**0.011**	45%
**High**	1	Jamalpur	0.095	8km	0.091	—	—	**C**	**0.029**	69%
	2	Sherpur	0.046	8km	0.044	—	—	**C**	**0.022**	52%
	3	Nasirabad	0.161	8km	0.149	—	—	**C**	**0.069**	57%
	4	Netrakona	0.055	8km	0.051	—	—	**C**	**0.022**	60%
	5	Tangail	0.138	1km	0.128	1km	0.137	**C**	**0.052**	62%
	6	Gazipur	0.304	5km	0.280	7km	0.301	**C**	**0.119**	61%
	7	Kishoreganj	0.162	1km	0.156	8km	0.161	**C**	**0.044**	73%
	8	Narshingdi	0.135	2km	0.119	1km	0.134	**C**	**0.042**	69%
	9	Manikgonj	0.192	1km	0.174	—	—	**C**	**0.031**	84%
	10	Dhaka	0.179	8km	0.156	1km	0.176	**C**	**0.045**	75%
	11	Naray Angonj	0.172	3km	0.130	1km	0.169	**C**	**0.039**	77%
	12	Munshigonj	0.083	8km	0.078	4km	0.082	**C**	**0.024**	71%
	13	Rajbari (west)	0.049	2km	0.045	—	—	**C**	**0.014**	71%
	14	Rajbari (east)	0.045	10km	0.039	—	—	**C**	**0.016**	64%
	15	Faridpur	0.059	8km	0.052	—	—	**C**	**0.017**	71%
	16	Gopalgonj	0.017	7km	0.016	—	—	**C**	**0.007**	59%
	17	Madaripur	0.059	1km	0.055	—	—	**C**	**0.014**	76%
	18	Shariatpur	0.020	2km	0.017	—	—	**C**	**0.007**	65%

Active surveillance strategy legend:
A - reactive by distance,
B - reactive by population,
C - proactive by population,
D - proactive by premises density,
Red. - Percentage decrease in probability of an epidemic using preferred intervention scheme compared to solely culling premises reporting infection.

Table F: Average outbreak duration for wave 5 type transmission dynamics under top performing scheme within each intervention type, stratified by the district where the outbreak originated. For each combination of capacity setting and district of outbreak origin (seed district), we present the average outbreak duration when only culling infected premises (IP cull only), alongside the top performing schemes for optimising this control objective within each of the three simulated intervention types (ring culling, ring vaccination, active surveillance). The strategy that optimised the objective (minimising the expected outbreak duration) is highlighted in bold. The mean duration values displayed were obtained by averaging over a minimum of 500 simulation runs. All duration values are given to 1 d.p. in units of days, with percentages given to 2 s.f.

	Seed district		Intervention type							
**Capacity**	**ID**	**Name**	**IP cull**	**Ring cull**		**Ring vacc.**		**Active surv.**		**Red.**
**Low**	1	Jamalpur	98.6	1km	94.7	7km	84.9	**C**	**61.6**	37%
	2	Sherpur	51.8	9km	50.3	9km	45.0	**C**	**36.2**	30%
	3	Nasirabad	159.1	1km	151.2	7km	136.9	**C**	**99.5**	37%
	4	Netrakona	59.6	10km	57.0	8km	52.1	**C**	**40.1**	33%
	5	Tangail	138.4	1km	128.0	7km	119.4	**C**	**87.2**	45%
	6	Gazipur	304.1	9km	278.7	8km	251.8	**C**	**168.6**	45%
	7	Kishoreganj	158.9	1km	152.6	9km	135.7	**C**	**87.6**	45%
	8	Narshingdi	135.7	1km	124.7	3km	115.8	**C**	**76.9**	43%
	9	Manikgonj	185.2	1km	172.8	7km	160.0	**C**	**77.3**	58%
	10	Dhaka	177.2	2km	161.0	4km	150.6	**C**	**77.3**	56%
	11	Naray Angonj	166.0	1km	146.6	1km	140.2	**C**	**83.3**	50%
	12	Munshigonj	87.1	1km	81.7	10km	74.6	**C**	**50.4**	42%
	13	Rajbari (west)	53.9	2km	50.8	8km	47.2	**C**	**28.5**	47%
	14	Rajbari (east)	51.0	9km	46.7	10km	44.7	**C**	**32.4**	37%
	15	Faridpur	62.5	9km	58.1	7km	54.7	**C**	**30.4**	51%
	16	Gopalgonj	23.6	10km	23.1	9km	21.1	**C**	**15.8**	33%
	17	Madaripur	63.6	1km	59.6	7km	55.7	**C**	**28.5**	55%
	18	Shariatpur	26.6	3km	24.2	9km	23.8	**C**	**17.8**	33%
**High**	1	Jamalpur	21.8	8km	19.8	6km	19.1	**C**	**9.0**	50%
	2	Sherpur	14.1	10km	13.2	10km	12.8	**C**	**8.8**	38%
	3	Nasirabad	29.8	10km	25.8	10km	25.4	**C**	**12.1**	59%
	4	Netrakona	15.4	10km	14.1	10km	13.8	**C**	**8.5**	45%
	5	Tangail	27.3	8km	24.2	7km	23.4	**C**	**10.9**	60%
	6	Gazipur	50.5	8km	41.7	9km	40.8	**C**	**15.8**	69%
	7	Kishoreganj	30.8	9km	27.5	8km	26.0	**C**	**10.0**	68%
	8	Narshingdi	27.7	3km	23.4	9km	23.4	**D**	**10.8**	61%
	9	Manikgonj	35.3	8km	29.9	10km	29.4	**C**	**8.8**	75%
	10	Dhaka	34.2	8km	28.2	10km	28.1	**C**	**10.4**	70%
	11	Naray Angonj	33.2	7km	24.9	9km	26.9	**C**	**10.3**	69%
	12	Munshigonj	20.1	8km	18.3	9km	17.5	**C**	**7.9**	61%
	13	Rajbari (west)	14.7	10km	13.5	10km	13.3	**C**	**8.1**	45%
	14	Rajbari (east)	14.3	10km	12.6	10km	12.9	**C**	**8.1**	43%
	15	Faridpur	16.2	9km	14.5	7km	14.4	**C**	**8.3**	49%
	16	Gopalgonj	9.5	7km	9.1	9km	9.0	**C**	**7.4**	22%
	17	Madaripur	16.3	4km	15.1	9km	14.5	**C**	**8.1**	50%
	18	Shariatpur	10.2	3km	9.5	10km	9.6	**C**	**7.6**	25%

Active surveillance strategy legend:

A - reactive by distance,
B - reactive by population,
C - proactive by population,
D - proactive by premises density,
Red. - Percentage decrease in average outbreak duration using preferred intervention scheme compared to solely culling premises reporting infection.

Table G: Average outbreak duration for wave 2 type transmission dynamics under different active surveillance strategies, stratified by the district where the outbreak originated. For each combination of capacity setting, district of outbreak origin (seed district) and active surveillance strategy the value displayed was obtained by averaging over 1,000 simulation runs. The strategy that optimises the objective (minimising the expected outbreak duration) is highlighted in bold. All duration values are given to 1 d.p. in units of days, with percentages given to 2 s.f.

	**Seed district**		**Active surveillance strategy**					
**Capacity**	**ID**	**Name**	**None**	**A**	**B**	**C**	**D**	**Red.**
**Low**	1	Jamalpur	14.9	14.8	14.7	**11.4**	14.5	24%
	2	Sherpur	14.5	14.4	14.3	**11.3**	13.5	22%
	3	Nasirabad	17.0	16.8	16.8	**12.3**	16.3	28%
	4	Netrakona	14.6	14.4	14.4	**11.5**	14.2	22%
	5	Tangail	16.8	16.4	16.4	**12.4**	15.7	26%
	6	Gazipur	19.5	19.3	19.3	**13.4**	18.2	31%
	7	Kishoreganj	17.1	16.7	16.7	**12.5**	16.4	27%
	8	Narshingdi	18.7	18.2	18.2	**13.8**	16.4	27%
	9	Manikgonj	16.7	16.3	16.2	**11.8**	15.9	29%
	10	Dhaka	17.6	17.0	16.8	**12.7**	15.7	28%
	11	Naray Angonj	18.1	17.4	17.2	**13.5**	14.1	26%
	12	Munshigonj	16.0	15.6	15.5	**12.0**	15.0	25%
	13	Rajbari (west)	14.3	14.3	14.3	**11.0**	13.9	23%
	14	Rajbari (east)	14.9	14.7	14.7	**11.4**	14.4	24%
	15	Faridpur	14.4	14.2	14.2	**10.9**	13.8	24%
	16	Gopalgonj	13.6	13.5	13.5	**10.6**	13.1	22%
	17	Madaripur	14.3	14.0	14.0	**10.5**	13.7	26%
	18	Shariatpur	14.3	14.1	14.1	**11.1**	13.7	22%
**Medium**	1	Jamalpur	14.9	14.7	14.7	**10.2**	13.9	32%
	2	Sherpur	14.5	14.3	14.3	**10.5**	12.1	28%
	3	Nasirabad	17.0	16.7	16.7	**11.4**	15.3	33%
	4	Netrakona	14.6	14.4	14.4	**10.6**	13.7	28%
	5	Tangail	16.8	16.4	16.4	**11.4**	15.1	32%
	6	Gazipur	19.4	19.1	19.1	**12.2**	16.6	37%
	7	Kishoreganj	17.0	16.6	16.6	**11.1**	15.5	35%
	8	Narshingdi	18.7	18.2	18.2	**12.9**	14.0	31%
	9	Manikgonj	16.7	16.2	16.2	**10.5**	14.9	37%
	10	Dhaka	17.5	16.8	16.8	**11.6**	14.5	34%
	11	Naray Angonj	18.0	17.3	17.2	**12.0**	13.2	33%
	12	Munshigonj	15.9	15.6	15.5	**10.3**	14.4	35%
	13	Rajbari (west)	14.3	14.2	14.2	**10.2**	13.4	29%
	14	Rajbari (east)	14.9	14.6	14.6	**10.3**	13.8	31%
	15	Faridpur	14.4	14.1	14.1	**10.2**	13.4	29%
	16	Gopalgonj	13.6	13.4	13.4	**9.9**	12.7	27%

Continued on next page

Table G: Continued from previous page

	Seed district		Active surveillance strategy					
**Capacity**	**ID**	**Name**	**None**	**A**	**B**	**C**	**D**	**Red.**
	17	Madaripur	14.2	14.0	14.0	**9.8**	13.3	31%
	18	Shariatpur	14.3	14.1	14.0	**10.3**	13.3	28%
**High**	1	Jamalpur	14.9	14.7	14.7	**8.4**	11.9	44%
	2	Sherpur	14.5	14.3	14.3	**9.2**	10.4	36%
	3	Nasirabad	16.9	16.6	16.6	**9.9**	12.9	42%
	4	Netrakona	14.6	14.3	14.3	**9.1**	12.4	37%
	5	Tangail	16.8	16.4	16.4	**9.5**	12.9	44%
	6	Gazipur	19.4	19.1	19.1	**9.9**	13.0	49%
	7	Kishoreganj	17.0	16.6	16.6	**8.8**	13.4	48%
	8	Narshingdi	18.7	18.1	18.1	10.5	**10.0**	46%
	9	Manikgonj	16.7	16.2	16.2	**8.2**	12.5	51%
	10	Dhaka	17.5	16.8	16.8	**9.2**	11.8	47%
	11	Naray Angonj	18.0	17.2	17.2	**9.8**	10.7	46%
	12	Munshigonj	15.9	15.5	15.5	**8.1**	13.1	49%
	13	Rajbari (west)	14.3	14.1	14.1	**9.1**	11.9	37%
	14	Rajbari (east)	14.8	14.6	14.6	**9.1**	12.6	39%
	15	Faridpur	14.4	14.1	14.1	**9.0**	12.1	37%
	16	Gopalgonj	13.6	13.4	13.4	**8.8**	11.6	35%
	17	Madaripur	14.2	13.9	13.9	**8.5**	12.0	40%
	18	Shariatpur	14.2	14.0	14.0	**9.2**	11.3	35%

Active surveillance strategy legend:
A - reactive by distance,
B - reactive by population,
C - proactive by population,
D - proactive by premises density,
Red. - Percentage decrease in average outbreak duration using preferred active surveillance scheme compared to when no active surveillance is imposed.

Table H: Epidemic probabilities for wave 2 type transmission dynamics under different active surveillance strategies, stratified by the district where the outbreak originated. For each combination of capacity setting, district of outbreak origin (seed district) and active surveillance strategy the value displayed was obtained by averaging over 1,000 simulation runs. The strategy that optimises the objective (minimising the probability of an epidemic) is highlighted in bold. All probabilities are given to 3 d.p., with percentages given to 2 s.f.

	Seed district		Active surveillance strategy					
**Capacity**	**ID**	**Name**	**None**	**A**	**B**	**C**	**D**	**Red.**
**Low**	1	Jamalpur	0.065	0.062	0.060	**0.012**	0.056	82%
	2	Sherpur	0.055	0.050	0.050	**0.010**	0.046	82%
	3	Nasirabad	0.103	0.098	0.098	**0.023**	0.092	78%
	4	Netrakona	0.065	0.059	0.059	**0.012**	0.060	82%
	5	Tangail	0.099	0.091	0.091	**0.024**	0.081	76%
	6	Gazipur	0.155	0.152	0.151	**0.039**	0.130	75%
	7	Kishoreganj	0.110	0.105	0.104	**0.022**	0.096	80%
	8	Narshingdi	0.138	0.130	0.130	**0.040**	0.108	71%

Continued on next page

Table H: Continued from previous page

	Seed district		Active surveillance strategy					
**Capacity**	**ID**	**Name**	**None**	**A**	**B**	**C**	**D**	**Red.**
	9	Manikgonj	0.096	0.086	0.085	**0.018**	0.078	81%
	10	Dhaka	0.111	0.104	0.102	**0.030**	0.082	73%
	11	Naray Angonj	0.122	0.115	0.112	**0.041**	0.073	66%
	12	Munshigonj	0.077	0.072	0.070	**0.018**	0.066	77%
	13	Rajbari (west)	0.058	0.056	0.054	**0.011**	0.051	81%
	14	Rajbari (east)	0.063	0.059	0.057	**0.013**	0.056	79%
	15	Faridpur	0.058	0.054	0.053	**0.009**	0.051	84%
	16	Gopalgonj	0.047	0.044	0.045	**0.008**	0.041	83%
	17	Madaripur	0.056	0.050	0.050	**0.005**	0.048	91%
	18	Shariatpur	0.056	0.052	0.052	**0.009**	0.051	84%
**Medium**	1	Jamalpur	0.065	0.059	0.060	**0.007**	0.047	89%
	2	Sherpur	0.055	0.050	0.050	**0.006**	0.035	89%
	3	Nasirabad	0.102	0.096	0.096	**0.011**	0.075	89%
	4	Netrakona	0.065	0.058	0.059	**0.006**	0.049	91%
	5	Tangail	0.099	0.091	0.091	**0.014**	0.067	86%
	6	Gazipur	0.155	0.150	0.150	**0.024**	0.100	85%
	7	Kishoreganj	0.110	0.103	0.103	**0.013**	0.079	88%
	8	Narshingdi	0.138	0.130	0.130	**0.025**	0.080	82%
	9	Manikgonj	0.096	0.085	0.085	**0.011**	0.066	89%
	10	Dhaka	0.110	0.101	0.101	**0.019**	0.066	83%
	11	Naray Angonj	0.122	0.114	0.112	**0.021**	0.058	83%
	12	Munshigonj	0.076	0.069	0.069	**0.008**	0.054	89%
	13	Rajbari (west)	0.058	0.054	0.054	**0.006**	0.043	90%
	14	Rajbari (east)	0.063	0.057	0.057	**0.005**	0.047	92%
	15	Faridpur	0.058	0.052	0.053	**0.005**	0.044	91%
	16	Gopalgonj	0.047	0.043	0.044	**0.003**	0.036	94%
	17	Madaripur	0.056	0.049	0.050	**0.003**	0.040	95%
	18	Shariatpur	0.056	0.052	0.052	**0.004**	0.042	93%
**High**	1	Jamalpur	0.065	0.059	0.059	**0.001**	0.024	98%
	2	Sherpur	0.055	0.050	0.050	**0.002**	0.018	96%
	3	Nasirabad	0.102	0.094	0.094	**0.002**	0.044	98%
	4	Netrakona	0.065	0.058	0.058	**0.001**	0.028	98%
	5	Tangail	0.099	0.091	0.091	**0.002**	0.036	98%
	6	Gazipur	0.154	0.149	0.149	**0.005**	0.047	97%
	7	Kishoreganj	0.109	0.103	0.103	**0.004**	0.047	96%
	8	Narshingdi	0.137	0.129	0.129	**0.008**	0.035	94%
	9	Manikgonj	0.096	0.085	0.085	**0.001**	0.038	99%
	10	Dhaka	0.110	0.101	0.101	**0.002**	0.037	98%
	11	Naray Angonj	0.121	0.112	0.112	**0.003**	0.026	98%
	12	Munshigonj	0.076	0.069	0.069	**0.001**	0.034	99%
	13	Rajbari (west)	0.058	0.053	0.053	**0.001**	0.031	98%
	14	Rajbari (east)	0.063	0.057	0.057	**0.001**	0.032	98%
	15	Faridpur	0.058	0.052	0.052	**0.001**	0.029	98%
	16	Gopalgonj	0.047	0.043	0.043	**0.001**	0.024	98%
	17	Madaripur	0.056	0.049	0.050	**0.001**	0.026	98%

Continued on next page

Table H: Continued from previous page

	Seed district		Active surveillance strategy					
**Capacity**	**ID**	**Name**	**None**	**A**	**B**	**C**	**D**	**Red.**
	18	Shariatpur	0.056	0.052	0.052	**0.001**	0.025	98%

Active surveillance strategy legend:
A - reactive by distance,
B - reactive by population,
C - proactive by population,
D - proactive by premises density,
Red. - Percentage decrease in epidemic probability using preferred active surveillance scheme compared to when no active surveillance is imposed.

Table I: Average outbreak duration for wave 5 type transmission dynamics under different active surveillance strategies, stratified by the district where the outbreak originated. For each combination of capacity setting, district of outbreak origin (seed district) and active surveillance strategy the value displayed was obtained by averaging over a minimum of 500 simulation runs. The strategy that optimises the objective (minimising the expected outbreak duration) is highlighted in bold. All duration values are given to 1 d.p. in units of days, with percentages given to 2 s.f.

	Seed district		Active surveillance strategy					
**Capacity**	**ID**	**Name**	**None**	**A**	**B**	**C**	**D**	**Red.**
**Low**	1	Jamalpur	98.6	95.5	95.4	**61.6**	96.5	37%
	2	Sherpur	51.8	51.6	51.6	**36.2**	49.4	30%
	3	Nasirabad	159.1	156.1	156.1	**99.5**	157.0	37%
	4	Netrakona	59.6	59.5	59.5	**40.1**	58.6	33%
	5	Tangail	138.4	132.5	132.5	**87.2**	131.4	45%
	6	Gazipur	304.1	282.5	282.4	**168.6**	282.2	45%
	7	Kishoreganj	158.9	156.8	156.7	**87.6**	152.8	45%
	8	Narshingdi	135.7	129.7	129.7	**76.9**	121.4	43%
	9	Manikgonj	185.2	177.2	177.2	**77.3**	177.3	58%
	10	Dhaka	177.2	165.0	165.1	**77.3**	149.5	56%
	11	Naray Angonj	166.0	138.3	136.4	**83.3**	106.0	50%
	12	Munshigonj	87.1	84.1	84.1	**50.4**	84.1	42%
	13	Rajbari (west)	53.9	52.9	52.9	**28.5**	53.9	47%
	14	Rajbari (east)	51.0	50.1	50.0	**32.4**	48.0	37%
	15	Faridpur	62.5	61.6	61.6	**30.4**	61.5	51%
	16	Gopalgonj	23.6	23.5	23.5	**15.8**	23.6	33%
	17	Madaripur	63.6	61.5	61.5	**28.5**	62.5	55%
	18	Shariatpur	26.6	25.6	25.6	**17.8**	26.6	33%
**Medium**	1	Jamalpur	43.4	41.9	41.9	**20.7**	41.9	52%
	2	Sherpur	24.6	24.3	24.3	**16.0**	22.4	35%
	3	Nasirabad	66.8	64.9	65.9	**38.0**	64.7	43%
	4	Netrakona	28.0	28.0	27.8	**15.6**	27.3	44%
	5	Tangail	58.9	56.3	56.3	**31.2**	54.5	47%
	6	Gazipur	118.9	118.7	117.7	**57.7**	113.5	52%
	7	Kishoreganj	66.6	67.6	67.1	**29.5**	61.0	56%
	8	Narshingdi	58.1	55.4	55.4	**28.8**	42.3	50%
	9	Manikgonj	78.0	74.0	73.7	**25.6**	69.4	67%

Continued on next page

Table I: Continued from previous page

	Seed district		Active surveillance strategy					
**Capacity**	**ID**	**Name**	**None**	**A**	**B**	**C**	**D**	**Red.**
	10	Dhaka	74.6	70.9	71.3	**29.5**	58.3	60%
	11	Naray Angonj	70.3	57.6	57.5	**31.3**	43.0	55%
	12	Munshigonj	39.0	37.5	37.5	**18.3**	35.9	53%
	13	Rajbari (west)	25.8	25.4	25.3	**12.4**	25.6	52%
	14	Rajbari (east)	24.7	24.2	24.2	**13.4**	23.4	46%
	15	Faridpur	29.4	28.8	28.8	**15.1**	28.7	49%
	16	Gopalgonj	13.6	13.5	13.5	**9.7**	13.5	28%
	17	Madaripur	29.8	28.8	28.8	**13.5**	29.2	55%
	18	Shariatpur	14.9	14.4	14.4	**9.9**	14.7	34%
**High**	1	Jamalpur	21.8	20.8	20.8	**9.0**	16.4	59%
	2	Sherpur	14.1	13.8	13.8	**8.8**	11.2	38%
	3	Nasirabad	29.8	28.4	28.4	**12.1**	23.6	59%
	4	Netrakona	15.4	15.1	15.1	**8.5**	14.0	45%
	5	Tangail	27.3	25.8	25.8	**10.9**	20.6	60%
	6	Gazipur	50.5	47.1	46.7	**15.8**	34.2	69%
	7	Kishoreganj	30.8	29.5	29.5	**10.0**	24.7	68%
	8	Narshingdi	27.7	26.1	26.1	**10.8**	14.4	61%
	9	Manikgonj	35.3	33.0	33.1	**8.8**	27.5	75%
	10	Dhaka	34.2	31.3	31.3	**10.4**	21.9	70%
	11	Naray Angonj	33.2	27.1	27.1	**10.3**	16.2	69%
	12	Munshigonj	20.1	19.2	19.2	**7.9**	17.1	61%
	13	Rajbari (west)	14.7	14.3	14.3	**8.1**	12.7	45%
	14	Rajbari (east)	14.3	13.9	13.9	**8.1**	12.9	43%
	15	Faridpur	16.2	15.7	15.7	**8.3**	14.6	49%
	16	Gopalgonj	9.5	9.4	9.4	**7.4**	9.1	22%
	17	Madaripur	16.3	15.7	15.7	**8.1**	14.6	50%
	18	Shariatpur	10.2	9.9	9.9	**7.6**	8.9	25%

Active surveillance strategy legend:
A - reactive by distance,
B - reactive by population,
C - proactive by population,
D - proactive by premises density,
Red. - Percentage decrease in average outbreak duration using preferred active surveillance scheme compared to when no active surveillance is imposed.

Table J: Epidemic probabilities for wave 5 type transmission dynamics under different active surveillance strategies, stratified by the district where the outbreak originated. For each combination of capacity setting, district of outbreak origin (seed district) and active surveillance strategy the value displayed was obtained by averaging over a minimum of 500 simulation runs. The strategy that optimises the objective (minimising the probability of an epidemic) is highlighted in bold. All probabilities are given to 3 d.p., with percentages given to 2 s.f.

	Seed district		Active surveillance strategy					
**Capacity**	**ID**	**Name**	**None**	**A**	**B**	**C**	**D**	**Red.**
**Low**	1	Jamalpur	0.095	0.093	0.093	**0.058**	0.093	39%

Continued on next page

Table J: Continued from previous page

	Seed district		Active surveillance strategy					
**Capacity**	**ID**	**Name**	**None**	**A**	**B**	**C**	**D**	**Red.**
	2	Sherpur	0.046	0.046	0.046	**0.030**	0.044	35%
	3	Nasirabad	0.161	0.157	0.157	**0.100**	0.159	38%
	4	Netrakona	0.055	0.055	0.055	**0.035**	0.054	36%
	5	Tangail	0.138	0.133	0.133	**0.088**	0.131	36%
	6	Gazipur	0.317	0.304	0.295	**0.176**	0.291	44%
	7	Kishoreganj	0.162	0.159	0.159	**0.090**	0.156	44%
	8	Narshingdi	0.135	0.129	0.129	**0.076**	0.121	44%
	9	Manikgonj	0.192	0.181	0.182	**0.080**	0.183	58%
	10	Dhaka	0.179	0.168	0.168	**0.075**	0.151	58%
	11	Naray Angonj	0.172	0.140	0.138	**0.091**	0.107	47%
	12	Munshigonj	0.083	0.081	0.081	**0.047**	0.080	43%
	13	Rajbari (west)	0.049	0.048	0.048	**0.022**	0.049	55%
	14	Rajbari (east)	0.045	0.044	0.044	**0.026**	0.042	42%
	15	Faridpur	0.059	0.058	0.058	**0.025**	0.058	58%
	16	Gopalgonj	0.017	0.017	0.017	**0.009**	0.017	47%
	17	Madaripur	0.059	0.057	0.057	**0.023**	0.058	61%
	18	Shariatpur	0.020	0.019	0.019	**0.011**	0.020	45%
**Medium**	1	Jamalpur	0.094	0.093	0.093	**0.040**	0.092	58%
	2	Sherpur	0.046	0.046	0.046	**0.026**	0.043	43%
	3	Nasirabad	0.161	0.157	0.159	**0.095**	0.158	41%
	4	Netrakona	0.055	0.055	0.055	**0.026**	0.054	53%
	5	Tangail	0.138	0.133	0.133	**0.073**	0.129	47%
	6	Gazipur	0.305	0.311	0.309	**0.156**	0.291	49%
	7	Kishoreganj	0.162	0.165	0.163	**0.068**	0.150	58%
	8	Narshingdi	0.135	0.129	0.129	**0.061**	0.098	55%
	9	Manikgonj	0.192	0.182	0.181	**0.054**	0.171	72%
	10	Dhaka	0.179	0.173	0.174	**0.065**	0.142	64%
	11	Naray Angonj	0.172	0.137	0.137	**0.073**	0.102	58%
	12	Munshigonj	0.083	0.081	0.081	**0.033**	0.077	60%
	13	Rajbari (west)	0.049	0.048	0.048	**0.015**	0.049	69%
	14	Rajbari (east)	0.045	0.044	0.044	**0.019**	0.042	58%
	15	Faridpur	0.059	0.058	0.058	**0.023**	0.058	61%
	16	Gopalgonj	0.017	0.017	0.017	**0.008**	0.017	53%
	17	Madaripur	0.059	0.057	0.057	**0.020**	0.058	66%
	18	Shariatpur	0.020	0.019	0.019	**0.080**	0.020	60%
**High**	1	Jamalpur	0.095	0.093	0.093	**0.029**	0.072	69%
	2	Sherpur	0.046	0.046	0.046	**0.022**	0.039	52%
	3	Nasirabad	0.161	0.157	0.157	**0.069**	0.135	57%
	4	Netrakona	0.055	0.055	0.055	**0.022**	0.052	60%
	5	Tangail	0.138	0.133	0.133	**0.052**	0.107	62%
	6	Gazipur	0.304	0.298	0.292	**0.119**	0.220	61%
	7	Kishoreganj	0.162	0.159	0.159	**0.044**	0.139	73%
	8	Narshingdi	0.135	0.129	0.129	**0.042**	0.070	69%
	9	Manikgonj	0.192	0.180	0.182	**0.031**	0.158	84%
	10	Dhaka	0.179	0.168	0.168	**0.045**	0.120	75%

Continued on next page

Table J: Continued from previous page

	Seed district		Active surveillance strategy					
**Capacity**	**ID**	**Name**	**None**	**A**	**B**	**C**	**D**	**Red.**
	11	Naray Angonj	0.172	0.137	0.137	**0.039**	0.078	77%
	12	Munshigonj	0.083	0.081	0.081	**0.024**	0.072	71%
	13	Rajbari (west)	0.049	0.048	0.048	**0.014**	0.042	71%
	14	Rajbari (east)	0.045	0.044	0.044	**0.016**	0.040	64%
	15	Faridpur	0.059	0.058	0.058	**0.017**	0.054	71%
	16	Gopalgonj	0.017	0.017	0.017	**0.007**	0.016	59%
	17	Madaripur	0.059	0.057	0.057	**0.014**	0.053	76%
	18	Shariatpur	0.020	0.019	0.019	**0.007**	0.016	65%

Active surveillance strategy legend:
A - reactive by distance,
B - reactive by population,
C - proactive by population,
D - proactive by premises density,
Red. - Percentage decrease in epidemic probability using preferred active surveillance scheme compared to when no active surveillance is imposed.

Table K: Predicted probability of outbreak duration t being 90 days or less for different active surveillance strategies. For each combination of transmission model, capacity restriction and active surveillance strategy the value displayed was obtained by averaging over a minimum of 500 simulation runs. For each model and control capacity combination the strategy that optimises the objective, having an outbreak duration below 90 days, is highlighted in bold. All values are given to 2 s.f.

		Active surveillance strategy				
**Transmission model**	**Control capacity**	**None**	**A**	**B**	**C**	**D**
**Wave 2**	Low	0.51	0.50	0.50	**0.55**	0.53
	Medium	0.51	0.50	0.50	**0.57**	0.55
	High	0.51	0.50	0.50	**0.61**	0.57
**Wave 5**	Low	0.38	0.40	0.40	**0.46**	0.38
	Medium	0.38	0.40	0.39	**0.48**	0.41
	High	0.38	0.39	0.39	**0.58**	0.47

Active surveillance strategy legend:
A - reactive by distance,
B - reactive by population,
C - proactive by population,
D - proactive by premises density.

Table L: Predicted probability of outbreak size *I* not exceeding 25 premises for different active surveillance strategies. For each combination of transmission model, capacity restriction and active surveillance strategy the value displayed was obtained by averaging over a minimum of 500 simulation runs. For each model and control capacity combination the strategy that optimises the objective, maximising the likelihood of having an outbreak with 25 premises or less infected, is highlighted in bold. All values are given to 2 s.f.

		**Active surveillance strategy**				
**Transmission model**	**Control capacity**	**None**	**A**	**B**	**C**	**D**
**Wave 2**	Low	0.69	0.70	0.70	**0.79**	0.72
	Medium	0.69	0.70	0.70	**0.82**	0.74
	High	0.69	0.70	0.70	**0.87**	0.81
**Wave 5**	Low	0.44	0.47	0.47	**0.52**	0.45
	Medium	0.44	0.46	0.46	**0.55**	0.48
	High	0.44	0.45	0.45	**0.64**	0.53

Active surveillance strategy legend:
A - reactive by distance,
B - reactive by population,
C - proactive by population,
D - proactive by premises density.

Table M: Mean number of poultry culled under different active surveillance strategies. For each combination of transmission model, capacity restriction and active surveillance strategy the value displayed was obtained by averaging over a minimum of 500 simulation runs. For each model and control capacity combination the strategy that optimises the objective, minimising the mean number of poultry culled, is highlighted in bold. All values are given to 2 s.f.

		**Active surveillance strategy**				
	**Capacity**	**None**	**A**	**B**	**C**	**D**
**Wave 2**	Low	3.0×10^5	2.8×10^5	2.7×10^5	$\mathbf{1.8 \times 10^5}$	2.6×10^5
	Medium	3.0×10^5	2.8×10^5	2.7×10^5	$\mathbf{1.5 \times 10^5}$	2.3×10^5
	High	3.0×10^5	2.7×10^5	2.7×10^5	$\mathbf{1.1 \times 10^5}$	1.7×10^5
**Wave 5**	Low	1.1×10^7	1.1×10^7	1.1×10^7	$\mathbf{0.8 \times 10^7}$	1.1×10^7
	Medium	1.0×10^7	1.0×10^7	1.0×10^7	$\mathbf{0.6 \times 10^7}$	0.9×10^7
	High	6.6×10^6	6.2×10^6	6.2×10^6	$\mathbf{1.7 \times 10^6}$	4.8×10^6

Active surveillance strategy legend:
A - reactive by distance,
B - reactive by population,
C - proactive by population,
D - proactive by premises density.

,

References

1. Food and Agriculture Organization of the United Nations. Approaches to controlling, preventing and eliminating H5N1 Highly Pathogenic Avian Influenza in endemic countries. FAO Anim Prod Heal Pap. 2011;(No. 171).
2. Biswas PK, Giasuddin M, Chowdhury P, Barua H, Debnath NC, Yamage M. Incidence of contamination of live bird markets in Bangladesh with influenza A virus and subtypes H5, H7 and H9. Transbound Emerg Dis. 2018;65(3):687–695. doi:10.1111/tbed.12788.
3. OIE. Highly pathogenic avian influenza, Bangladesh, (Follow-up report No. 43). OIE, WAHID(World Anim Heal Inf Database), Wkly Dis Inf. 2013;26(52).
